# Protein O‐Glycosylation Shapes Lymphatic Endothelia and Lymph Node Macrophage Populations

**DOI:** 10.1096/fj.202600240R

**Published:** 2026-09-11

**Authors:** Jasmin Frey, Annkathrin Ratter, Finn Brigger, Louie Cielen, Pascal Kunz, Ea Tulin, Kinga Mikos, Lothar Dieterich, Marco D'Addio, Cornelia Halin, Richard D. Cummings, Vivianne I. Otto

**Affiliations:** ^1^ Institute of Pharmaceutical Sciences ETH Zurich Zurich Switzerland; ^2^ Department of Surgery, Beth Israel Deaconess Medical Center Harvard Medical School Boston Massachusetts USA

**Keywords:** cell surface glycoproteins, glycome, lymphatic endothelia, macrophages, murine lymph node, O‐glycosylation

## Abstract

Endothelial cells were among the first cells identified to have a surface glycocalyx, but its functions are still elusive. Within the glycocalyx are many glycoproteins whose O‐glycosylation is partly regulated by *Cosmc*, an essential chaperone for functional activity of T‐synthase that adds galactose to O‐glycans initiating their elongation. Using mice with inducible deletion of either *Cosmc (C1Galt1C1)* or the sialyltransferase *St3Gal‐1* in lymphatic endothelial cells (LEC), we discovered that loss of elongated O‐glycans is accompanied by major changes in glycoprotein composition, including podoplanin, CD44, Lyve‐1, and ICAM‐1. These changes in the endothelial glycocalyx are sensed by adhesion molecules, such as Siglec‐1, and lead to major changes in the size and phenotype of lymph node macrophage populations. Our findings demonstrate the crucial roles of O‐glycosylation in regulating LEC functions and lymph node macrophage differentiation.

## Graphical abstract

To study the functional roles of O‐glycans on lymphatic endothelia, we deleted Cosmc, the chaperone required for core‐1 O‐glycan synthesis. Loss of core‐1 O‐glycans entailed major changes in glycoprotein composition of lymphatic endothelial cells (LEC), including reduced podoplanin (Pdpn), CD44, and Lyve‐1, and increased ICAM‐1; reduced CD44 was caused by cleavage to a C‐terminal fragment. When induced in LEC of newborn mice, the loss of core‐1 O‐glycans entailed major changes in the size and phenotype of lymph node macrophage populations.

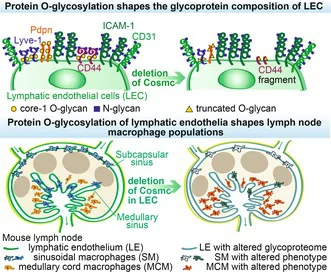

## 1 Introduction

The mammalian vasculature consists of hierarchically organized blood and lymphatic vessels. On their luminal side, lymphatic vessels are lined by lymphatic endothelial cells (LEC) that express the lineage‐determining transcription factor Prospero homeobox protein‐1 (Prox1) [1]. LEC also have a unique glycocalyx which contains many glycoproteins, including the lymphatic endothelial hyaluronan receptor‐1 (Lyve‐1) and podoplanin, a heavily O‐glycosylated glycoprotein essential for the development and maintenance of the lymphatic vascular system [2, 3, 4]. Platelet endothelial cell adhesion molecule‐1 (PECAM‐1/CD31) is expressed by both blood and lymphatic vascular endothelial cells. Among the LEC in lymph nodes (LN), there are three major cell subsets—LEC lining the subcapsular sinus ceiling (cLEC) and floor (fLEC), and the medullary sinus (mLEC)—that exhibit substantial heterogeneity in terms of gene expression [5, 6, 7, 8]. Among these, the fLEC stand out as they display a unique glycocalyx characterized by particularly high densities of sialylated O‐glycans linked to Ser/Thr in glycoproteins [9].

fLEC and mLEC closely interact with the sinusoidal macrophages situated in the subcapsular sinus (SSM; Siglec‐1^+^ F4/80^−^ SIGNR‐1^−^) and the medullary sinus (MSM; Siglec‐1^+^ F4/80^+^ SIGNR‐1^+^). The sinusoidal macrophages are directly exposed to the incoming lymph from which they scavenge antigens and pathogens and pass them on to the lymphocytes in the LN parenchyma [10]. SSM capture and translocate viral particles and immune complexes to B cells and lipid antigens to natural killer T cells [11, 12, 13, 14].

The interactions between sinusoidal macrophages and LEC are mediated by adhesion molecules such as the macrophage integrin Mac‐1 (CD11b/CD18) and its ligand intercellular adhesion molecule‐1 (ICAM‐1/CD54) on LEC. They also involve binding of specific glycans within the endothelial glycocalyx by the multiple glycan binding proteins (GBP) expressed by macrophages, such as Siglec‐1 (sialoadhesin/CD169) which interacts with sialylated O‐glycans [15, 16], and SIGN‐R1 which recognizes oligomannose and some fucosylated N‐glycans [17]. In addition to the LN sinus, the LN parenchyma contains specific macrophage populations; The medullary cord macrophages (MCM; Siglec‐1^−^ F4/80^+^ SIGNR‐1^−^) reside in the medullary cords adjacent to the medullary sinus. They are thought to provide the plasma blasts and plasma cells emerging upon immunization with trophic support. They are also involved in clearing plasma cells that undergo apoptosis after the peak of the primary immune response [10].

fLEC are a key component of the cellular niche for SSM, producing the signaling factors necessary for the establishment, homeostatic maintenance, and restoration of the SSM population after inflammation [18, 19]. We have postulated that the cellular niche for SSM additionally depends on the uniquely dense α2,3‐sialylated O‐glycans expressed by fLEC [9]. In the LN of mice whose macrophages carry a non‐functional Siglec‐1 mutant, SSM were less abundant and displayed a less proinflammatory phenotype. This suggested that the α2,3‐sialylated O‐glycans on fLEC may be involved in interactions with GBP such as Siglec‐1 expressed by macrophages, and thus support their presence and shape their phenotype in the LN.

To directly define the potential roles of glycoprotein O‐glycans we targeted their biosynthetic pathway. A master regulator of this pathway is the endoplasmic reticulum molecular chaperone Cosmc (*C1Galt1C1*), encoded by an X‐linked gene; its function is to regulate expression of the single client enzyme T‐synthase (*C1Galt1*). T‐synthase is a unique Golgi enzyme that initiates elongation of O‐glycans in all cells by adding galactose (Gal) to N‐acetylgalactosamine (GalNAc) and thus generates the universal core 1 O‐glycan (Galβ1,3‐GalNAcα‐Ser/Thr). This structure is typically modified to a sialylated form and one of the enzymes known to generate α2,3‐sialylated O‐glycans is ST3Gal‐1. Cosmc regulates folding of nascent T‐synthase and expression of the functional enzyme; loss or impairment of Cosmc function leads to inactivity of T‐synthase, a collapse of O‐glycosylation, and consequent accumulation of truncated O‐glycans, primarily the Tn antigen GalNAcα‐Ser/Thr.

In the present study, we assessed the functional roles played by sialylated O‐glycans in the LN sinuses, with the goal of elucidating the role of such glycans (i) in shaping the glycocalyx of the sinusoidal LEC, and (ii) in the formation and maintenance of macrophage populations in the mouse LN. The results identify novel and essential roles of O‐glycans in regulating the glycoprotein composition of the LEC glycocalyx and the postnatal formation of the macrophage populations in the lymph node.

## 2 Animals, Materials and Methods

### 2.1 Mice

*Cosmc* ^*ΔLEC*^ mice were generated by crossing *Prox1*‐Cre^ERT2^ mice with Cosmc^fl/fl^/Cosmc^Y/fl^ mice (kindly provided by Prof. Taija Mäkinen, Uppsala University, Sweden and Prof. Richard D. Cummings, Department of Surgery, Beth Israel Deaconess Medical Center, Harvard Medical School, Boston Massachusetts, USA, respectively). *Prox1*‐Cre^ERT2^ *St3Gal1* ^fl/fl^ mice were generated by crossing *Prox1*‐Cre^ERT2^ mice with *St3gal1* ^fl/fl^ mice [20]. To induce Cosmc or St3Gal1 deficiency in pups, Cre^+^ and Cre^−^ littermates were injected with tamoxifen (Sigma‐Aldrich; dissolved in sunflower oil) intra‐gastrically at P1‐P3 (25 μL at 2 mg/mL). Additionally, Cosmc deficiency was induced in adult mice at 8 to 9 weeks of age by intraperitoneal tamoxifen injection on five consecutive days (1 mg/20 g body weight). These protocols have been optimized previously to induce stable gene deletions in LEC while minimizing stress for the animals by using the lowest effective tamoxifen doses and numbers of daily injections [21]. All analyses were performed in adult mice (at least 8 weeks of age). The mice with adult onset of Cosmc deletion were analyzed 4 weeks after the last tamoxifen injection, a time span considered sufficient for tamoxifen clearance, establishment of Cosmc deletion, turnover of the LEC glycocalyx and adaptation of sinusoidal macrophage populations in the lymph node [18]. All mice used in this study were housed in our OHB facility, had food and water ad libitum and a 12 h light/dark cycle at constant temperature (22°C) and humidity (55%). In all experiments, animals from both sexes were used when comparing between the different genotypes. All experiments were approved by the Cantonal Veterinary Office Zurich under Project Licenses ZH005/18, ZH040/22, and ZH173/25.

### 2.2 Murine Lymph Node Lymphatic Endothelial Cell Isolation and Culture

Isolation of primary murine lymph node LEC was performed as previously described (Commerford, Dieterich et al. 2018) [22] one to two weeks after induction of *Cosmc* or *St3Gal1* deletion in adult mice. In brief, skin‐draining LN were digested with 0.25 mg/mL Liberase DH (Roche) and 200 U/mL DNAse I (Roche) in RPMI medium (Gibco/Thermo Fisher) for 1 h at 37°C. The LN single cell suspension was strained through a 70 μm filter and resuspended in MEM‐alpha medium (Gibco) supplemented with 100 U/mL penicillin/streptomycin, 10% FCS and 2 mM L‐glutamine for culturing in pre‐coated cell culture dishes (10 μg/mL PureCol collagen solution Type I and 10 μg/mL fibronectin; Merck Millipore) at 37°C and 5% CO_2_. At confluency, cells were detached by using Accutase (Biological Industries, Beit HaEmek, Israel) and ECs were positively selected with CD31‐conjugated microbeads (Miltenyi Biotec. Bergisch Gladbach, Germany). LEC were used at passage three to five for experiments.

### 2.3 CORA Method to Analyze *O‐*Glycome of LN LEC

The Cellular O‐glycome Reporter/Amplification (CORA) method was performed as previously described [23]. First, acetylation required to obtain the cell penetrable Ac_3_GalNAc‐α‐Bn (CORA‐reporter), was performed by adding freshly prepared pyridine: acetic anhydride (2:1; V/V) to benzyl 2‐acetamido‐2‐deoxy‐α‐d‐galactopyranoside (Bn‐α‐GalNAc; Sigma‐Aldrich, Cat#B4894). The mixture was stirred for 90 min at 65°C and complete triacetylation of the compound was confirmed by LC–MS analysis. Next, the organic solvents were evaporated by SpeedVac (Eppendorf) and the obtained sticky residue was resuspended in water, frozen for 1 h at −80°C and lyophilized overnight. The dried product was resolved in DMSO to make a 50 mm stock solution. The purity of the CORA‐reporter was checked by LC–MS. Second, 4 × 10^5^ LN LEC were seeded in a 60.1 cm^2^ dish at passage four. After four days, the cells had reached approximately 70% of confluency an the medium was replaced by fresh medium containing 50 μm CORA‐reporter. After three days, the media was collected, centrifuged and the supernatant was filtered over 10‐kDa centrifugal filter (Amicon Ultra 10 K, Merck) at 3500 rpm for 30 min. The flow‐through containing Bn‐O‐glycans was loaded onto a C18 Sep‐Pak column (200 mg, Waters) equilibrated with 0.1% trifluoracetic acid (v/v). After four washes with 2 mL 0.1% trifluoracetic acid (v/v), Bn‐O‐glycans were eluted with 50% (v/v) acetonitrile, 0.1% (v/v) trifluoracetic acid. To remove organic solvents, the eluate was concentrated by SpeedVac (Eppendorf), resuspend in water and lyophilized. Third, Bn‐O‐glycans were permethylated using standard procedures and analyzed on the Bruker Ultraflextreme MALDI‐MS instrument.

### 2.4 Extraction of Glycosphingolipids, N‐Glycans and O‐Glycans

Cultured, primary LN LEC were harvested using Accutase and the cell pellet (∼4.5 * 10^6^ cells) was washed extensively with DPBS, followed by snap freezing in liquid nitrogen. To extract glycosphingolipids (GSL), the samples were dissolved in chloroform: methanol (2:1 v/v). Following 10 min sonication and centrifugation at 6000 *g* for 5 min, the supernatant was collected to prepare extracted GSL, and the pellet was used for N‐ and O‐glycan analysis as described below.

To purify the GSL from the supernatant, a slurry containing NaOH in DMSO was added to dried samples, followed by the addition of 200 μL of methyl iodide (Merck). After 20 min mixing at room temperature, the reaction was terminated by drop‐wise addition of water. When the reaction stopped boiling, 2 mL of chloroform was added to the samples. The water layer was removed after liquid–liquid extraction. The procedure of chloroform‐water extraction was repeated twice. Finally, the chloroform layer was removed under a stream of nitrogen. GSL were permethylated using standard procedures and analyzed on the Bruker Ultraflextreme MALDI‐MS instrument.

For sample preparation of N‐ and O‐glycans, the lysate was incubated with 1 μL benzonase nuclease (250 units, Sigma‐Aldrich) for 30 min at 37°C to degrade DNA and thereby reduce sample viscosity. Next, 100 μL of 25 mM iodoacetamide were added for 1 h in the dark for alkylation. The lysates were centrifuged for 30 min at 6000 *g* and the supernatant was transferred to a fresh tube. 100 μL of 20% trichloroacetic acid (TCA) was added following heavy vortexing. The samples were kept on ice for 40 min, before the supernatant was removed. The pellet was washed three times with 100 μL acetone, followed by drying the proteins using Speed‐Vac. The subsequent tryptic digest was performed by adding 0.1 μg of trypsin in 25 mM ammonium bicarbonate (ABC) buffer (pH 8.5) for overnight incubation at 37°C. The mixture was then centrifuged for 30 min at 6000 *g* and trypsin in the supernatant was deactivated by boiling at 95°C for 10 min. To release *N‐*glycans, samples were digested by the addition of 1 μL PNGase F (New England Biolabs, 500 000 units/mL) and incubated overnight at 37°C. The next day, additional 1 μL PNGase F was added, and the sample was incubated for another 6 h. The released *N‐*glycans were separated from the (glyco)peptides using Sep‐Pak C18 (Waters) and a Porous Graphitic Carbon (PGC) solid phase extraction cartridge (Thermo Fisher). The PGC cartridge was washed with 2% ACN and *N‐*glycans were eluted using 25% ACN followed by concentration on the Speed‐Vac, permethylation and analysis at the Bruker Ultraflextreme MALDI‐TOF‐TOF.

The de‐*N‐*glycosylated (glyco)peptides (de‐*N‐*peptides) were eluted from Sep‐Pak C18 by 20% isopropanol with 5% acetic acid and 40% isopropanol with 5% acetic acid, collected in glass tubes and dried. To release O‐glycans, the 𝛽‐elimination reaction was performed as previously described [24]. In brief, de‐*N‐*peptides were incubated with 400 μL borohydride solution (1 M sodium borohydride in 0.05 M NaOH) at 45°C for 14–16 h. The reaction was terminated by the dropwise addition of concentrated acetic acid on ice, until the fizzing stopped. The sample was loaded directly onto a prepared Dowex column (50 W‐X8 50–100 mesh, BioRad), eluted with twice the column volume of 5% acetic acid and dried by Speed‐Vac. The borates were removed by repeated addition of 10% acetic acid in methanol to the sample and dried under a stream of nitrogen, followed by permethylation and MALDI‐MS analysis.

### 2.5 RNA Extraction

LN LEC at confluency were washed twice with DPBS, and cell lysis and RNA isolation were performed using the NucleoSpin RNA kit (Macherey‐Nagel, Düren, Germany) following the manufacturer's instructions. Reverse transcription was performed using the High‐Capacity cDNA Transcription kit (Thermo Fisher Scientific) following the manufacturer's instructions.

### 2.6 qRT‐PCR

Quantitative PCR analyses on extracted RNA from cultured cells were performed on the 7900HT Fast Real‐Time PCR System (Thermo Fisher Scientific) using PowerUp SYBR Green Mastermix and the following primers to analyze gene expression of Cosmc (C1galt1c1 F: 5′‐GAA AGC AGT TCC TTT TTG AAG GG‐3′; R: 5′‐GGT GGT GCA TTC TGT TTC CAA‐3′), St3gal1 (St3gal1 F: 5′‐TCC CAA AGT GAC CCT GTG TCT CTG‐3′: R_WT: 5′‐ATG TGA AGA CAC AGG TGA CTG CCA‐3′; R_KO: 5′‐CGG TAC CCG GGG ATC AAT TCG AG‐3′), Lyve‐1 (F: 5′‐CAG CAC ACT AGC CTG GTG TTA‐3′; R: 5′‐CGC CCA TGA TTC TGC ATG TAG A‐3′), podoplanin (F: 5′‐TGC CAG TGT TGT TCT GGG TT‐3′; R: 5′‐AGC CCT CCA GTA GCA CCT GT‐3′), CD31 (F: 5′‐CCA AAG CCA GTA GCA TCA TGG TC‐3′; R: 5′‐GGA TGG TGA AGT TGG CTA CAG G‐3′), CD44 (F: 5′‐AGC AGC GGC TCC ACC ATC GAG A‐3′; R: 5′‐TCG GAT CCA TGA GTC ACA GTG‐3′), VCAM‐1 (F: 5′‐TGA ACC CAA ACA GAG GCA GAG T‐3′; R: 5′‐GGT ATC CCA TCA CTT GAG CAG G‐3′), ICAM‐1 (F: 5′‐CAA TTT CTC ATG CCG CAC AG‐3′; R: 5′‐AGC TGG AAG ATC GAA AGT CCG‐3′), and Gapdh (F: 5′‐CCT GGA GAA ACC TGC CAA GTA TG‐3′; R: 5′‐AGA GTG GGA GTT GCT GTT GAA GTC‐3′).

### 2.7 Flow Cytometry of Cultured, Primary LN LECs

Cells were detached from culture dishes using Accutase and washed twice with flow cytometry buffer (1% FCS, 2 mM EDTA and 0.02% sodium azide in PBS). To characterize cell surface expression of podoplanin (PDPN), CD31, CD44, VCAM‐1, and ICAM‐1, LN LECs were incubated with anti‐PDPN—PE antibody (1:400; 8.1.1, eBioscience), anti‐CD31‐FITC (1:300; BD 553372), anti‐CD44‐APC antibody (1:200; rat IgG2a, IM7, binding an extracellular epitope located between amino acids 145 and 186; BD 559259), anti‐VCAM‐1‐FITC (1:500; rat IgG2a, 429, Biolegend 105 705), and anti‐ICAM‐1‐PE (1:200; YN1/1.7.4., Biolegend 116 107), respectively. To phenotype cell surface glycosylation and assess binding of Siglec‐1, the cells were incubated with either biotinylated lectins, namely MAL‐II ( *Maackia amurensis* lectin‐II, 20 μg/mL), PNA (peanut agglutinin, 10 μg/mL; both from Vector Laboratories, Burlingame, CA, USA), and HPA ( *Helix pomatia* , 20 μg/mL; Sigma‐Aldrich), or Siglec‐1‐Fc (100 μg/mL in PBS; produced and purified as previously described (D'Addio, Frey et al. 2021)) [9] for 30 min on ice. Bound plant lectins and Siglec‐1‐Fc were visualized with either fluorescein‐streptavidin (Vector Laboratories) or DyLight488 secondary goat anti‐human IgG Fc (Invitrogen). The cell viability was assessed by Zombie NIR (1:500, BioLegend). To determine non‐specific binding, cells were incubated with fluorescein‐streptavidin or secondary antibody alone. Before acquisition, the cells were washed and resuspended in flow cytometry buffer.

Flow cytometric analysis was performed on a Beckman Coulter CytoFLEX flow cytometer. Data was analyzed using the FlowJo version 10.5.3 software. The delta geometric mean fluorescence intensities (ΔgMFI) were calculated as the difference between the “specific staining” obtained using both the primary and secondary staining agents (biotinylated lectin and streptavidin‐PE or primary antibody and fluorescently labeled secondary antibody or Siglec‐1‐Fc and fluorescently labeled anti‐Fc antibody) and the “non‐specific binding” from the secondary staining agent alone (streptavidin‐PE (SA‐PE) or fluorescently labeled secondary antibody or fluorescently labeled anti‐Fc antibody).

### 2.8 Western Blot Analysis of Cell Lysates of Cultured, Primary LN LEC

Cells were washed twice with PBS and detached from culture dishes using Accutase. They were lysed in cold lysis buffer (1% TritonX (T8787, Sigma Aldrich), 150 mM NaCl (A7006, AppliChem), 50 mM TrisHCl (T3038, Sigma‐Aldrich), and protease and phosphatase inhibitor cocktail (11 697 498 001, Roche)). Lysates were kept at 4°C for 1 h and were vigorously vortexed every 15 min for 10 s. They were centrifuged, and the protein concentration was determined in the supernatant using the Pierce BCA Protein Assay Kit (A55865, Thermo Fisher Scientific).

To strip proteins of their glycans (and at the same time assess the type of glycans present) 10 μg of total protein were treated with various combinations of sialidase (α2‐3,6,8 Neuraminidase; P0720L, New England Biolabs), O‐glycanase (P0733L, New England Biolabs), α‐N‐Acetyl‐galactosaminidase (P0734S, New England Biolabs), and PNGase F (P0704S, New England Biolabs) as indicated in the Figures. Samples were incubated with PNGase F and sialidase alone for 1 h at 37°C following the manufacturer's protocol. α‐N‐Acetylgalactosaminidase and O‐Glycosidase were used in combination with sialidase in 1× Glyco Buffer 1 (B1727S, New England Biolabs) and incubated with the cell lysates overnight at 37°C. Before addition of the glycosidases requiring such prolonged incubations, the samples were boiled at 95°C for 5 min to inactivate any proteases. The reactions were terminated by placing the samples on ice.

Samples were incubated with 4× Laemmli sample buffer (161‐0747, Bio‐Rad) containing 10% β‐mercaptoethanol (M6250, Thermo Fisher Scientific) and boiled at 95°C for 5 min. 10 μg of total protein was loaded per well. SDS‐PAGE was performed using either 4%–20% (M00656, GenScript; for analysis of CD44) or 4%–12% gradient gels (M00653, GenScript; for analysis of ICAM‐1). In addition, 5 μL of the Pre‐Stained Protein Ladder (Precision Plus Protein Dual Color Standards, Bio‐Rad #1610374) were loaded into the first and last well of each gel. Gels were run using the GenBox Mini Electrophoresis System (GenScript) at 50 mV for 30 min, 110 mV for 1 h 15 min. Proteins were then transferred to either a 0.45 μm or a 0.2 μm nitrocellulose membrane (for analysis of ICAM‐1 and CD44, respectively; 1 620 146 and 1 620 145, BioRad) at 16 V for 20 min using the Bio‐Rad Trans‐blot semi‐dry electrophoretic transfer cell.

The membranes were dried for 45 min and stained with Revert 700 Total Protein Stain (926‐11 011, LI‐COR) following the manufacturer's protocol to control for comparable protein loading. The fluorescence signals were recorded using the ChemiDoc MP Imaging System (734BR2553, BioRad).

Membranes were then incubated in 5% milk/TBS‐Tween 0.1% for 90 min, followed by overnight incubation at 4°C with the primary antibody against ICAM‐1 (AF796, R&D Systems) or CD44 (ab157107, Abcam; rabbit polyclonal antibody directed against peptide within human CD44 corresponding to aa 692‐742 at the C‐terminus) diluted 1:1000 in 5% milk/TBS‐Tween 0.1% on a rocking platform shaker. Following three 10 min washes in TBS‐T, the membranes were incubated with horseradish peroxidase (HRP)‐conjugated rabbit anti‐goat antibody (61‐1620, Thermo Scientific) or donkey anti‐rabbit antibody (A‐16035, Thermo Scientific) diluted 1:1000 in 5% milk/TBS‐Tween 0.1% at room temperature in the dark for 3 h. Membranes were washed three times in TBS‐Tween 0.1% for 10 min, incubated with the chemiluminescence substrate (1 705 061, Bio‐Rad) for 5 min in the dark at room temperature and imaged using the ChemiDoc MP Imaging System (734BR2553, BioRad).

### 2.9 Immunofluorescence of Frozen Mouse LN Sections

Skin‐draining LN were dissected, embedded in optimum cutting temperature compound (OCT, Tissue‐Tek, Sysmex Suisse, Horgen, Switzerland), and snap frozen in liquid nitrogen. Frozen sections of 7 μm thickness were cut and fixed with acetone (−20°C) and 80% methanol (4°C, room temperature). After fixation, sections were washed with TBS‐T (TBS with 0.2% Tween 20) and blocked with blocking solution (either a combination of 5% donkey serum with 1% BSA or 5% BSA alone in TBS‐T) for 1 h at room temperature. If the staining involved biotinylated lectins, this step was replaced by incubation with Carbo‐Free Blocking solution (Vector Laboratories), followed by a second blocking step using the Streptavidin/Biotin Blocking Kit (Vector Laboratories) according to the manufacturer's instructions.

The sections were incubated with primary antibodies or lectins for 2 h at room temperature or overnight at 4°C as follows: O6 rat IgG (in house reagent [25]; 20 μg/mL in TBS‐T), rat anti‐mouse B220 (clone RA3‐6B2, 1:100; BD), Armenian hamster anti‐mouse CD31 (clone 2H8, 1:250–1:300; Thermo Fisher), rat anti‐mouse CD31 (clone MEC 13.3, 1:200; BD Pharmingen or clone 2H8, Invitrogen), polyclonal goat anti‐mouse Lyve‐1 (1:200; R&D), rat anti‐mouse CD11b (clone M1/70, 1:100; BD), Syrian hamster anti‐mouse podoplanin (clone 8.1.1, 1:150; Abcam), rat anti‐mouse CD44 (clone W19210B, 1:200; BioLegend), goat anti‐mouse VCAM‐1 (5 μg/mL; R&D), goat anti‐mouse ICAM‐1 (5 μg/mL; R&D), rat anti‐mouse Siglec‐1 (clone 3D6.112, 1:100; Adserotec), rat anti‐mouse SIGNR‐1 (clone 22D1, 1:100; BioCell), rat anti‐mouse F4/80 (clone Cl:A3‐1, 1:100; BioRad), biotinylated MAL‐II (2 μg/mL; Vector Laboratories), biotinylated HPA (2.5 μg/mL; Sigma‐Aldrich), biotinylated PNA (12.5 μg/mL; Vector Laboratories). After washing, bound antibodies were visualized with secondary polyclonal antibodies labeled with Alexa Fluor 488, Alexa Fluor 594, or Alexa Fluor 647 (1:200, all Invitrogen, Carlsbad, CA if not mentioned otherwise), namely donkey anti‐goat, goat anti‐rat, donkey anti‐rat (Abcam), goat anti‐Armenian hamster, goat anti‐Syrian hamster. Bound biotinylated lectins were visualized with streptavidin conjugated with Alexa Fluor 488 (1:100, S‐32354) or Alexa Fluor 594 (1:200, S‐32356). Slides were counterstained with Hoechst 33342 (1:1000; Invitrogen), washed and mounted with either Mowiol mounting medium or ProLong gold (9071S, Cell Signaling Technology). Images were acquired using either a confocal Zeiss LSM 780 upright microscope with a Zeiss AxioImager. Z2 (ETH Zurich) or a Zeiss LSM 880 upright or inverted live cell confocal system (Confocal Imaging Core, BIDMC, Harvard, Boston, MA). Whole lymph node scans were acquired using an Olympus VS200 Slide Scanner at the Neurobiology Imaging Facility (NIF) at Harvard Medical School, Boston, MA.

### 2.10 Flow Cytometry Analysis of LN Immune Cells

The analysis of LN immune cells was performed as previously described [9]. In brief, single mouse LN or two pooled auricular LN from the same mouse were chopped using scissors and digested in 750 μL basic medium supplemented with 0.4 mg/mL collagenase IV (Gibco Thermo Fisher) and 40 μg/mL DNAse I (Roche) at 37°C on a rotating wheel for 25 min. An automated multichannel pipette (Eppendorf Xplorer plus) was used to disaggregate the remaining LN fragments by pipetting up and down 99 times, 7.5 μL of 0.5 m EDTA were added to maintain the single cell suspension and the pipetting cycles were repeated. An equal volume of basic medium was added, and the single cell suspension was strained through a 40 μm cell strainer, washed with flow cytometry buffer and pelleted by centrifugation. If two LN had been pooled, the total cells were divided into two fractions and each of them was transferred to a round bottom 96‐well flow cytometry plate. To block non‐specific antibody binding, the cells were incubated with CD16/32 (1:50, BioLegend) in PBS on ice for 20 min. Then, the cells were incubated for 30 min on ice with the primary antibodies (all from BioLegend, if not mentioned otherwise). One of the two LN single cell fractions was incubated with the antibodies CD11b—BV605 (1:100, M1/70), F4/80—AF647 (1:100, CI:A3‐1, Bio‐Rad), Siglec‐1 (CD169)—PE‐Cy7 (1:100, 3D6.112), SIGNR‐1—BV421 (1:200, ER‐TR9, BD), CX3CR1—PE (1:400, SA011F11), CD80—BV650 (1:50, 16‐10A1), and the viability dye Zombie Aqua (1:500) in PBS. In addition, CD3—FITC (1:200, 17A2), CD19—FITC (1:200, 6D5) and LY6‐G—FITC (1:400, 1A8, BD Biosciences) antibodies were used to dump out leukocytes [26]. To measure proliferation, intracellular staining was performed with Ki67—eFluor450 (1:200, SoIA15, eBioscience, San Diego, CA, USA) using an intracellular staining kit (72–5775, eBioscience) according to the manufacturer's instructions. The second LN fraction was incubated with CD3—PE‐Cy7 (1:200, 145‐2C11), B220—APC (1:200, RA3‐6B2), CD19—FITC and Zombie Aqua. Cells were resuspended in flow cytometry buffer and analyzed on a LSR Fortessa cell analyzer, flow cytometric data were analyzed using the FlowJo version 10.5.3 software.

### 2.11 Statistical Analyses

The statistical significance of differences between mean values was calculated using a paired or unpaired, two‐tailed Student's *t*‐test at a confidence level of 95% as indicated in the Figure legends. Differences were considered statistically significant when *p* < 0.05. All statistical analyses were performed using the GraphPad Prism software versions 9.3.1 and 10.4.1.

## 3 Results

### 3.1 Loss of Cosmc/T‐Synthase Function Causes Loss of α2,3‐Sialylated O‐Glycans on Lymph Node Lymphatic Endothelial Cells

**FIGURE 1:**
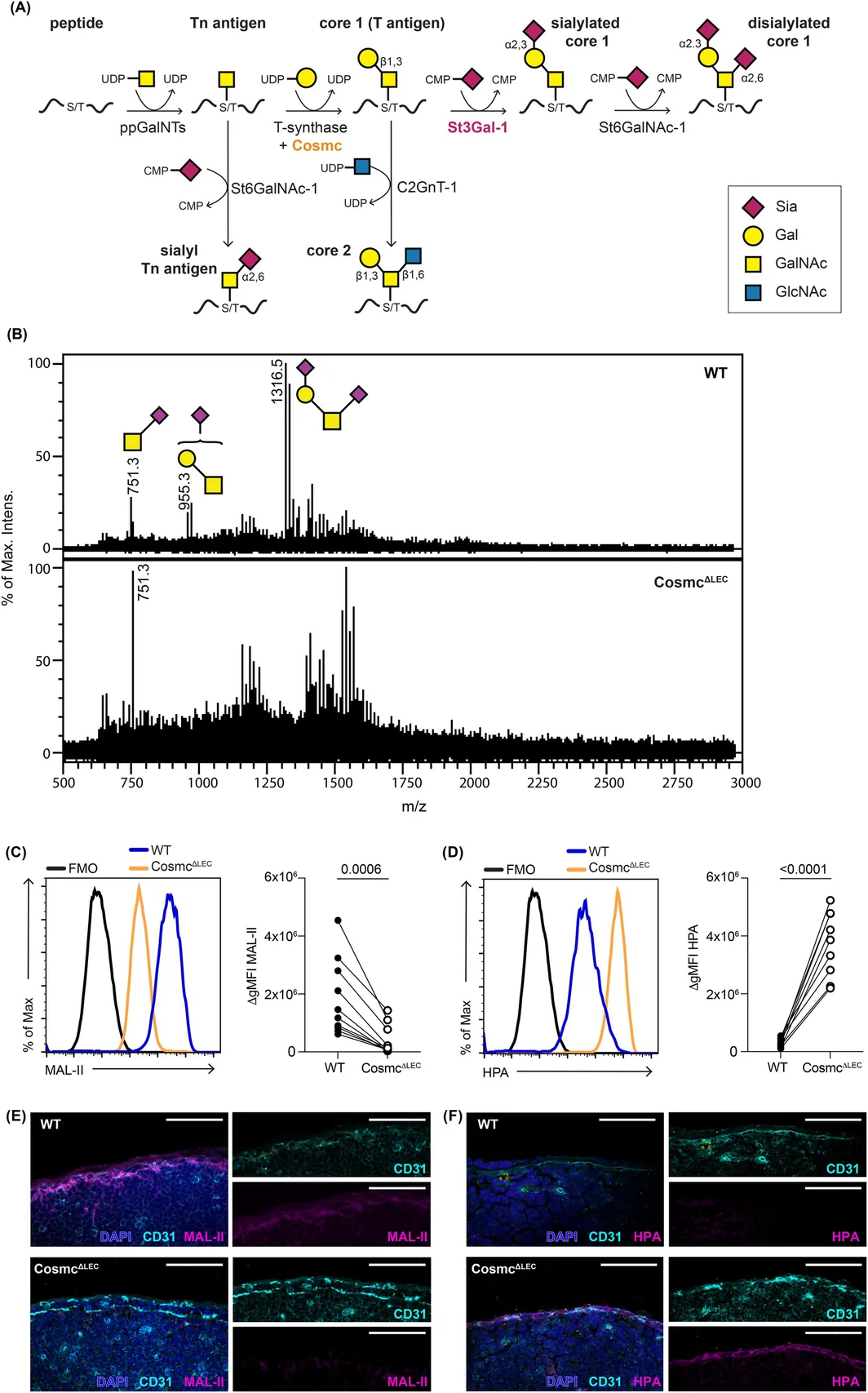
Loss of Cosmc/T‐synthase function causes a loss of α2,3‐sialylated O‐glycans on lymph node lymphatic endothelial cells. (A) In the O‐glycosylation pathway, T‐synthase is the only enzyme capable of adding galactose (Gal) in 𝛽1,3‐linkage to the *N‐*acetylgalactosamine (GalNAc) on serine or threonine (S/T). To be active, T‐synthase requires the molecular chaperone Cosmc. The core 1 O‐glycan is sialylated by the sialyltransferases St3Gal‐1 (adding sialic acid (Sia) in α2,3‐linkage to Gal) and St6GalNAc‐1 (adding sialic acid in α2,6‐linkage to GalNAc). The T‐antigen may also be modified by core 2 GlcNAc transferase (C2GnT‐1) to form the core 2 O‐glycan. The symbols used to depict glycans follow the Symbol Nomenclature for Glycans (SNFG) (Varki, Cummings et al. 2015) [27]. (B) MALDI‐TOF MS of permethylated benzyl (Bn)‐O‐glycans produced by cultured, primary lymph node lymphatic endothelial cells (LN LEC) that have been incubated with the CORA reporter (Ac_3_GalNAc‐α‐Bn). The cultured LN LEC were derived from Cosmc^ΔLEC^ and wild type (WT) littermate controls. Peaks are scaled relative to the maximum signal intensity. Putative glycan structures matching the detected masses are shown next to the corresponding peak. Flow cytometric analysis of (C) MAL‐II ( *Maackia amurensis* lectin‐II) and (D) HPA ( *Helix pomatia* agglutinin) binding to cultured, primary LN LEC from WT and *Cosmc* ^*ΔLEC*^ mice. LN LEC stained omitting the lectin were used as negative FMO (fluorescence minus one) controls. Representative flow cytometric histograms (left) and summary of the delta geometric mean fluorescence intensities (ΔgMFI) of 10 independent experiments. Data deriving from the same experiment are connected by a line. Paired Student's *t*‐test. (E, F) Binding of MAL‐II and HPA to LN sections of 8‐week‐old WT and *Cosmc* ^*ΔLEC*^ mice that had been treated with tamoxifen postnatally (compare Figure 3A), counter‐stained for cell nuclei (DAPI) and endothelial cells (CD31). Scale bars are 50 μm.

To assess the functional roles of α2,3‐sialylated O‐glycans on LEC, we tested two strategies to block essential steps in their synthesis: the initiation of core 1 O‐glycan elongation by T‐synthase and the addition of sialic acid by the sialyltransferase St3Gal‐1 (Figure 1A). There are two α2,3sialyltransferases that can sialylate the core 1 O‐glycan—St3Gal‐1 and St3Gal‐2 [28]. Because the murine St3Gal‐1 is thought to have more activity toward core 1 O‐glycans than St3Gal‐2, we chose to target *St3Gal‐1* [29]. We thus generated mice with inducible LEC‐specific losses of either *Cosmc* (*C1Galt1C1*) (*Cosmc* ^*ΔLEC*^ mice) or *St3Gal‐1* (*St3gal1* ^*ΔLEC*^ mice) (Figures S1A and S2A). To assess O‐glycosylation in LEC, we cultured LN LEC isolated from adult *Cosmc* ^*ΔLEC*^ mice and used the CORA method, in which cells are incubated with an O‐glycan precursor (peracetylated benzyl‐GalNAc/Ac_3_GalNAc‐α‐Bn). This precursor is incorporated by cells and then used as a substrate by all the enzymes in the O‐glycan biosynthetic pathway, to generate the cellular repertoire of O‐glycans which are secreted into culture media (Figure S1B) [23]. We observed that the LN LEC derived from *Cosmc* ^*ΔLEC*^ mice expressed the sialyl Tn antigen, but not the larger mono‐ or di‐sialylated core 1 O‐glycans, which were produced by the LEC from wild‐type mice (Figure 1B). To validate and specify this finding, we explored binding of *Maackia amurensis* lectin‐II (MAL‐II), which recognizes α2,3‐sialylated and 3‐O‐sulfated Galβ1,3‐GalNAc. MAL‐II binding was virtually abolished and binding of *Helix pomatia* agglutinin (HPA), which recognizes the Tn antigen, was concomitantly increased in cultured LN LEC and SCS fLEC in sections of mouse LN (Figure 1C–F). Thus, both the cultured LEC derived from mouse LN and the LEC in the LN tissue of *Cosmc* ^*ΔLEC*^ mice had reduced the synthesis of α2,3‐sialylated core 1 O‐glycans considerably and expressed the Tn/sialyl Tn antigen instead. Loss of *Cosmc* led to a strong reduction in binding of Siglec‐1 which is known to have preferential binding to α2,3‐sialylated O‐glycans (Figure S1C) [15], but did not affect the N‐glycan or glycosphingolipid (GSL) profiles of cultured LN LEC. Among the N‐glycans, the sialylated, complex type *N‐*glycans represented only a minor fraction whereas oligomannose structures predominated (Figure S1D). Using a specific monoclonal antibody to 3‐O‐sulfated glycans (O6; [25]), we observed that these were abundantly expressed on the fLEC of both WT and *Cosmc* ^ΔLEC^ mice (Figure S1E,F). The major types of GSL were GM3 and the non‐sialylated LC_4_/GB_4_Cer, whereas the disialylated gangliosides GD3 and GD1a/GD1b represented minor species (Figure S1G). Among the O‐glycans synthesized by cultured LN LEC derived from *St3gal1* ^*ΔLEC*^ mice, there was a clear increase in the relative amounts of non‐sialylated O‐glycans compared to WT LN LEC as detected by mass spectrometry and staining by peanut agglutinin (PNA; binds to non‐sialylated core 1 O‐glycans; Figure S2B–F). However, there were also significant residual amounts of sialylated structures—probably formed by the compensatory action of other sialyltransferases such as St3Gal‐2—as seen by mass spectrometry and residual staining by MAL‐II. Taken together, loss of *Cosmc* expression, while significantly reducing elongated O‐glycans and Siglec‐1 ligands, did not affect the N‐glycan or GSL profiles of LEC. Because of the profound effect of *Cosmc* deletion on biosynthesis of α2,3‐sialylated O‐glycans compared to that observed for *St3gal1* ^*ΔLEC*^ mice, we focused further studies on *Cosmc* ^ΔLEC^ mice.

### 3.2 Loss of Cosmc/T‐Synthase Function Affects the Abundances of Prominent Glycoproteins on Lymphatic Endothelial Cells in Various Ways

**FIGURE 2:**
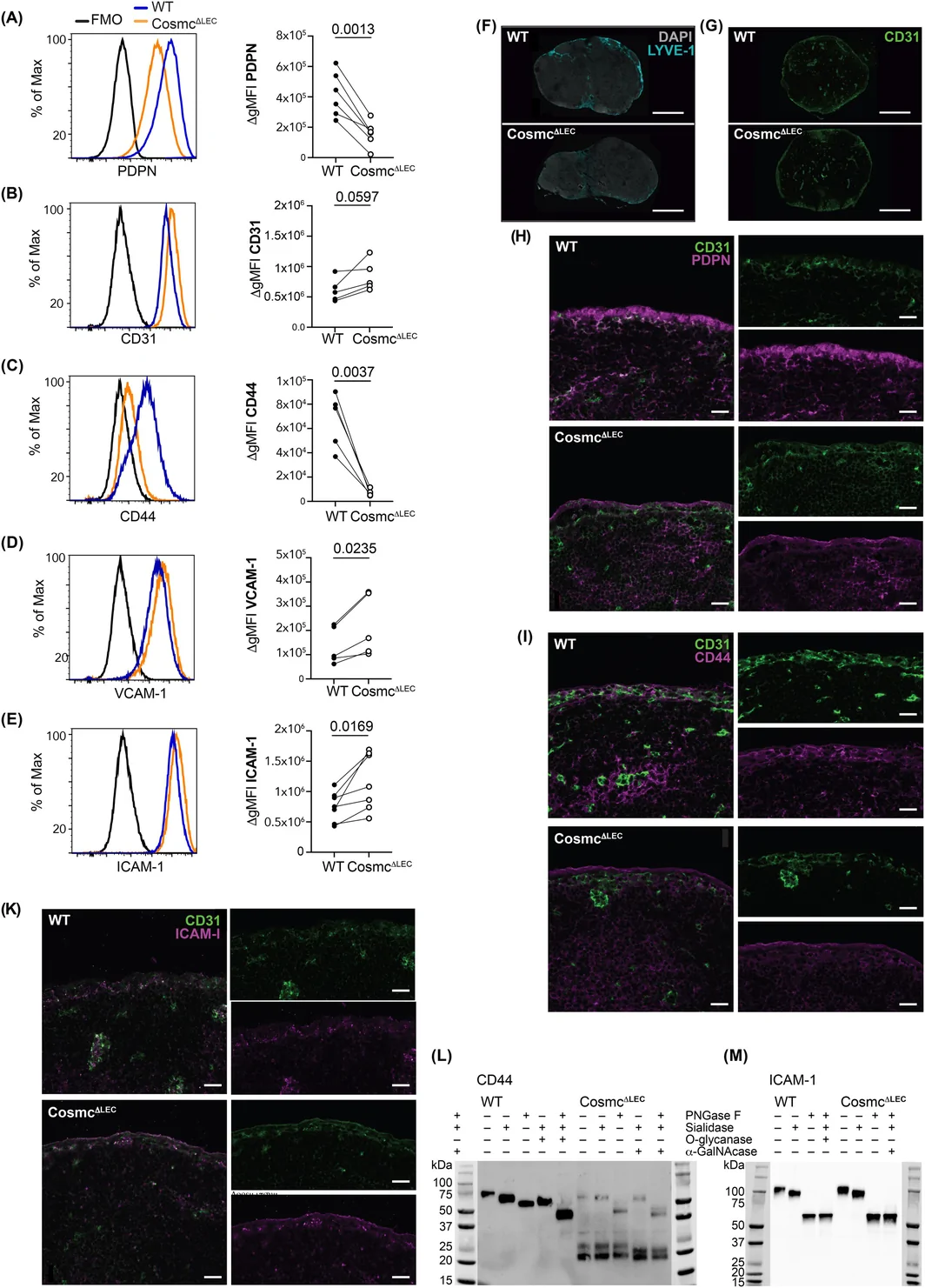
Loss of Cosmc/T‐synthase function affects the abundances of prominent glycoproteins on lymphatic endothelial cells in various ways. Cultured primary LN LEC from tamoxifen‐treated Cre+ and Cre− animals were analyzed by flow cytometry for the expression of (A) podoplanin/PDPN, (B) CD31, (C) CD44, (D) VCAM‐1, and (E) ICAM‐1. The antibody used for detection of CD44 binds to an N‐terminal epitope on CD44. LN LEC stained omitting the primary antibody were used as negative FMO (fluorescence minus one) controls. Representative flow cytometric histograms (left) and summary of the delta geometric mean fluorescence intensities (ΔgMFI) of 5–7 independent experiments (right) are shown. Data deriving from the same experiment are connected by a line. Paired Student's *t*‐test. The LN of Cre+ and Cre− animals that had been treated with tamoxifen postnatally were analyzed 8 weeks later for the expression of (F) Lyve‐1 and (G) CD31 by immunofluorescence. Abundance of LYVE‐1 (a protein marker for LEC), but not of CD31 (a pan endothelial cell marker) was strongly reduced on the lymphatic endothelia forming the LN subcapsular sinus of *Cosmc* ^*ΔLEC*^ mice. CD31 was thus used for localization of LEC in the analysis of (H) podoplanin/PDPN, (I) CD44, and (K) ICAM‐1 expression. Representative images from 3 independent experiments are shown. Scale bars are 500 μm (F, G) and 20 μm (H, I, K). Cell lysates of cultured LN LEC from WT and *Cosmc* ^*ΔLEC*^ mice were analyzed for CD44 (L) and ICAM‐1 (M) by Western blot prior (−) and after (+) treatment with the deglycosylating enzymes PNGase F (cleaving N‐glycans), sialidase (cleaving sialic acid), and O‐glycanase (cleaving desialylated core‐1 O‐glycans) or α‐GalNAcase (cleaving terminal α‐GalNAc and thus the desialylated Tn antigen). The antibody used for detection of CD44 binds to a membrane‐proximal epitope of CD44. To estimate molecular weights a ladder of proteins with known molecular weights (given in kDa) was run. Representative images from two biological replicates are shown.

Based on a previous report on loss of podoplanin expression in *Cosmc*‐deficient LEC [30], we explored whether the deletion of *Cosmc* might affect the expression of podoplanin and other prominent membrane glycoproteins in LN LEC. Flow cytometric analysis of cultured LEC isolated from WT and *Cosmc* ^*ΔLEC*^ mouse LN showed a significant reduction in the amounts of podoplanin (Figure 2A) and CD44 (Figure 2C). By contrast, there were slight increases in the abundance of VCAM‐1 (Figure 2D) and ICAM‐1 (Figure 2E), but no significant change in the abundance of CD31 (Figure 2B). *Immunofluorescence analysis* revealed that LYVE‐1 was strongly reduced in the LN of *Cosmc* ^*ΔLEC*^ mice compared to WT (Figure 2F), whereas CD31 and ICAM‐1 were hardly altered (Figure 2G,K). Podoplanin and CD44 levels were reduced on the CD31^+^ endothelial cells in the LN subcapsular sinus (Figure 2H,I). Interestingly, no changes in podoplanin, CD44, VCAM‐1, ICAM‐1, or Lyve‐1 levels were observed in the LN LEC of *St3gal1* ^*ΔLEC*^ mice (Figure S2G–M). The change in glycoprotein abundance in cultured, primary LEC of *Cosmc* ^*ΔLEC*^ mice was not due to altered levels of the pertaining transcripts as assessed by qPCR (Figure S3) and confirmed by RNA sequencing.

An alternative regulatory mechanism may be posttranslational proteolysis and ectodomain shedding of glycoproteins with truncated O‐glycans [31]. To explore this possibility, we analyzed CD44 and ICAM‐1 in lysates of WT and *Cosmc* ^*ΔLEC*^ LEC by Western blot; both CD44 and ICAM‐1 may be released from cell surfaces by proteolysis [32, 33]. We found that CD44 from LN LEC carried N‐ and O‐glycans (as revealed by molecular weight shifts upon digestion with PNGase F and sialidase/O‐glycanase) whereas ICAM‐1 carried only N‐glycans (as revealed by a large shift in molecular weight upon PNGase F treatment, but not upon sialidase/O‐glycanase treatment; Figure 2L,M). Using an antibody that binds an epitope close to the C‐terminus of CD44, we detected one to three CD44 bands differing in molecular weight. A high molecular weight form at ca. 75 kDa was N‐ and O‐glycosylated and low molecular weight forms at 20–25 kDa were only O‐glycosylated. The high molecular weight CD44 was abundant in WT LEC lysates but only present in trace amounts in *Cosmc* ^*ΔLEC*^ LEC (Figure 2L); the low molecular weight forms of CD44 predominated in *Cosmc* ^*ΔLEC*^ LEC. Previous studies on human CD44 have reported on CD44 C‐terminal fragments of 20–25 kDa resulting from proteolysis by the metalloprotease ADAM 10 [32, 34]. Thus, the reduced CD44 levels observed on *Cosmc* ^*ΔLEC*^ LEC by FACS analysis (Figure 2C) were most likely due to increased proteolysis to a C‐terminal fragment of CD44 that was not detectable by the antibody used. In contrast, ICAM‐1 appeared as one N‐glycoprotein of 75–90 kDa that was equally abundant in WT and *Cosmc* ^*ΔLEC*^ LEC (Figure 2M). Taken together, deletion of *Cosmc* and the ensuing loss of function of T‐synthase led to significant changes in the glycoprotein composition of LEC. The heavily O‐glycosylated proteins podoplanin, Lyve‐1, and CD44 were reduced in their abundance, whereas levels of the N‐glycosylated Ig‐like adhesion molecules VCAM‐1 and ICAM‐1 were slightly increased. The heavily N‐glycosylated CD31 (PECAM‐1) was hardly altered in its abundance. The changes in glycoprotein composition of the LEC surface upon loss of extended O‐glycans were primarily due to altered levels of heavily O‐glycosylated glycoproteins and arose through post‐transcriptional mechanisms such as increased proteolysis.

### 3.3 Loss of Cosmc/T‐Synthase Activity in Lymphatic Endothelial Cells After Birth Affects the Formation and Phenotypes of Sinusoidal Macrophage Populations in the Mouse Lymph Node

**FIGURE 3:**
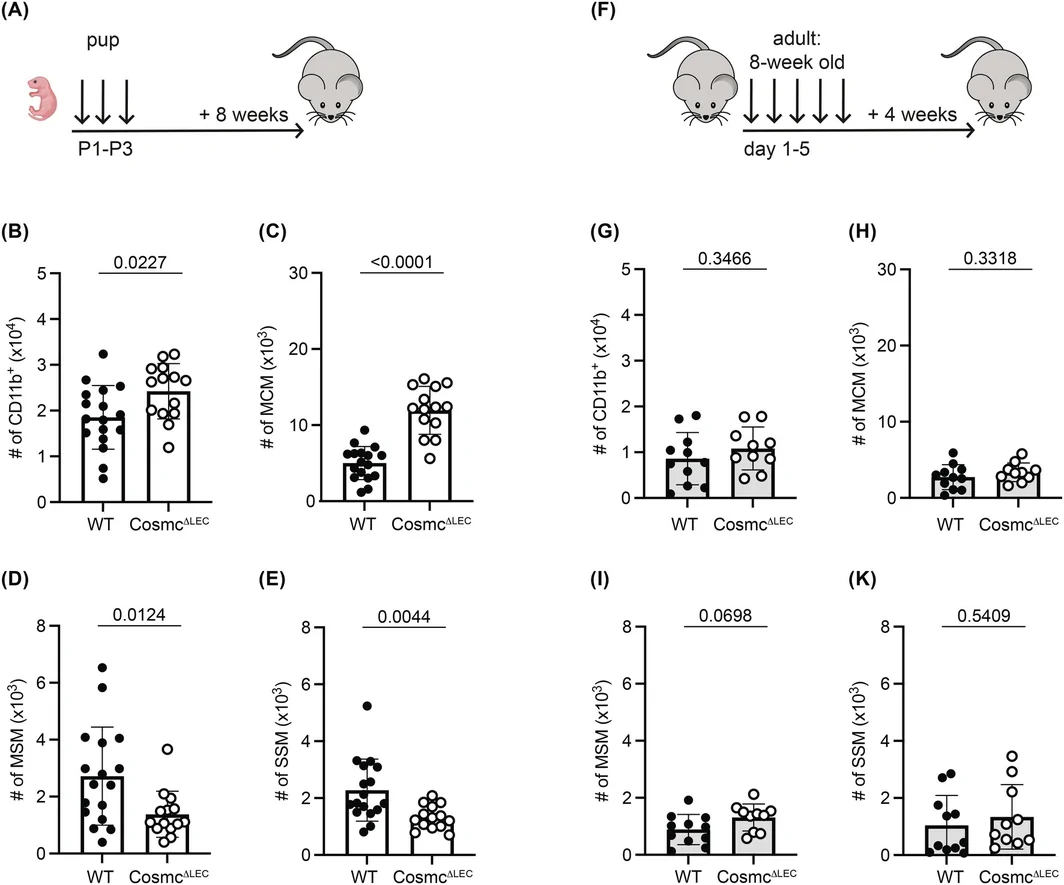
Loss of Cosmc/T‐synthase activity in lymphatic endothelial cells after birth affects the formation of macrophage populations in the mouse lymph node. Two experimental set‐ups were used in treating mice with tamoxifen to delete the floxed allele of *Cosmc* in *Prox1*‐expressing lymphatic endothelial cells (LEC). (A) Pups were treated with tamoxifen on three consecutive days (arrows) starting at postnatal day (P) 1 and lymph nodes (LN) were analyzed 8 weeks later. (F) Alternatively, mice were treated with tamoxifen on five consecutive days (arrows) when 8 weeks old and analyzed 4 weeks later. Cre^−^ littermates served as wild type (WT) controls. The auricular LN of adult *Cosmc* ^*ΔLEC*^ and WT mice treated using either the experimental set‐up (A) or (F) were analyzed by flow cytometry. The numbers (#) of all CD11b^+^ LN macrophages (B, G) and of the medullary cord macrophages (MCM; C, H), medullary sinus macrophages (MSM, D, I), and subcapsular sinus macrophages (SSM; E, K) are shown. The data derive from 3 pooled experiments (B–E; *n*_WT_ = 17, *n*_*CosmcΔLEC*_ = 14) and 2 pooled experiments (G–K; *n*_WT_ = 11, *n*_*CosmcΔLEC*_ = 10) and are given as single as well as mean values ±SD. Unpaired Student's *t*‐test.

We then asked whether the altered LEC glycoproteome upon *Cosmc* deletion in either newly born or adult mice might affect the macrophage populations in the mouse LN. It has been reported that after birth, BM‐derived monocytes immigrate into LN and then proliferate to establish the adult SSM network [19]. We therefore induced loss of *Cosmc* in newly born mice and quantified their LN macrophages by flow cytometry, when they had reached adulthood (Figure 3A, for gating strategy see Figure S4A). There was a 30% increase in the number and frequency of CD11b^+^ cells in the LN of *Cosmc* ^*ΔLEC*^ mice as compared to *Cre* ^−^ controls (Figure 3B, Figure S4B). This increase appeared to be due to a considerably larger population of MCM (2‐fold increase on average in numbers and percentage of CD11b^+^ LN macrophages; Figure 3C; Figure S4C) in combination with reduced numbers of the less abundant sinusoidal macrophage subsets, MSM and SSM (reduced on average by 40%–60%; Figure 3D,E, Figure S4D,E).

Next, we induced loss of *Cosmc* in 8 weeks‐old adult mice and quantified the LN macrophages 4 weeks later (Figure 3F). There were no significant changes in the numbers of total CD11b^+^ cells, MCM, MSM, or SSM in *Cosmc* ^*ΔLEC*^ mice compared to WT mice (Figure 3G–K). This suggests that the glycoproteome of LEC shaped by *Cosmc* and T‐synthase activity is not required for the maintenance of LN macrophage populations in adult mice. Loss of *Cosmc* in either new‐born or adult mice had no significant impact on the LN weights, LN cellularity, numbers of T and B cells or organization of the lymph nodes (Figure S5B–E,G–K). The LNs of the mice with postnatal loss of *Cosmc*, however, tended to be heavier and redder (Figure S5B,M). This observation may suggest some spilling of blood into the lymphatic vessels of these mice.

**FIGURE 4:**
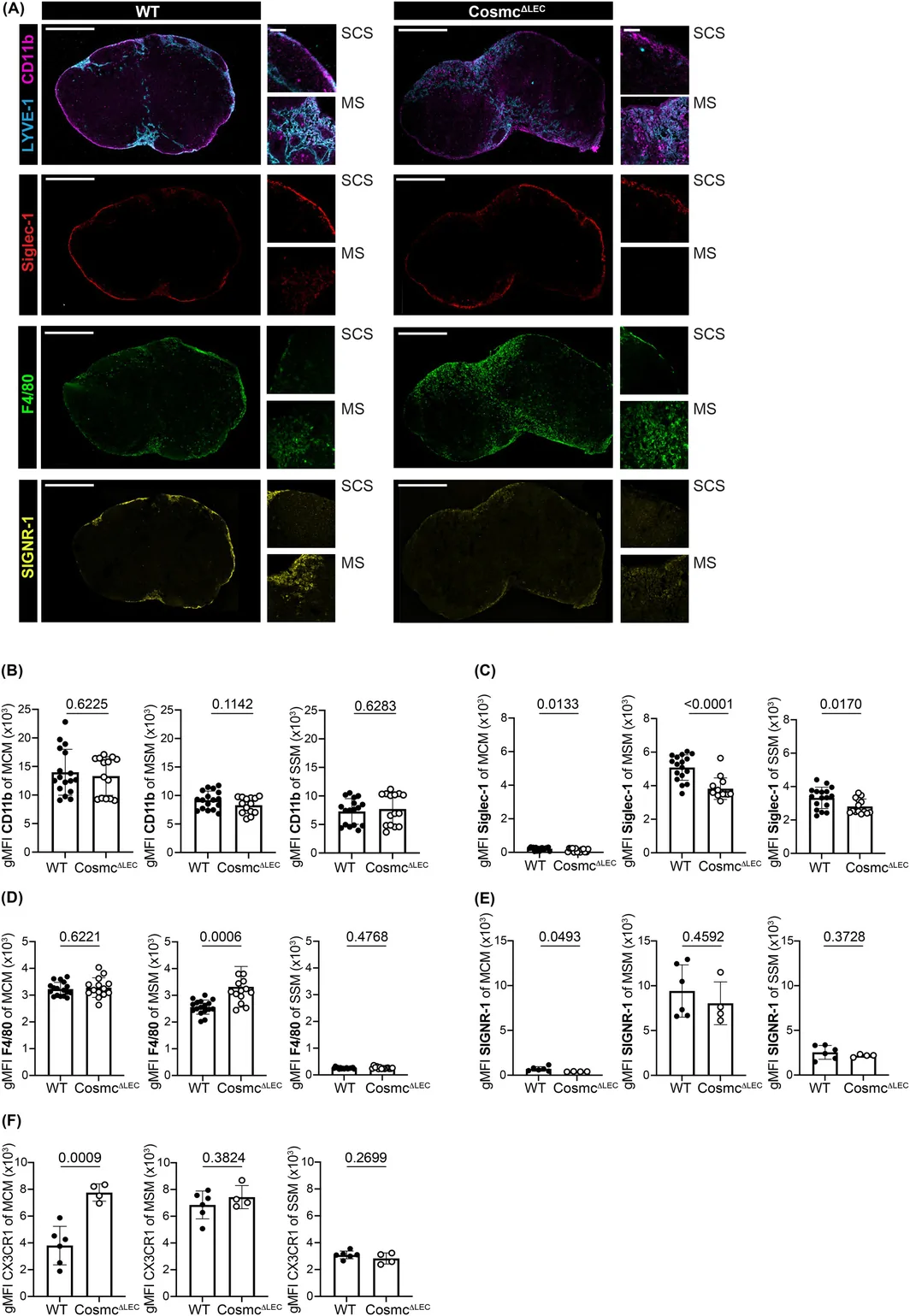
Loss of Cosmc/T‐synthase activity in lymphatic endothelial cells after birth affects the phenotypes of macrophage populations in the mouse lymph node. (A) Consecutive sections of auricular LN from adult WT and *Cosmc* ^*ΔLEC*^ mice that had been treated with tamoxifen postnatally were analyzed for expression of CD11b, Siglec‐1, F4/80, and SIGNR‐1 (by LN macrophages). Shown are the whole LN (left, scale bar = 500 μm) and details of the subcapsular sinus (SCS) and medullary sinus (MS) (right, scale bar = 50 μm). Lyve‐1 expression highlights lymphatic endothelia in the LN of WT mice (top panel) but is very weak in the LN of *Cosmc* ^*ΔLEC*^ mice (compare Figure 2F). Expression of CD11b (B), Siglec‐1 (C), F4/80 (D), SIGNR1 (E), and CX3CR1 (F) by medullary cord macrophages (MCM), medullary sinus macrophages (MSM), and subcapsular sinus macrophages (SSM) isolated ex vivo from the auricular lymph nodes of adult WT and *Cosmc* ^*ΔLEC*^ mice that had been treated with tamoxifen postnatally was measured by flow cytometry (3 pooled experiments with *n*_WT_ = 17, *n*_*CosmcΔLEC*_ = 14 (B–D) and two pooled experiments with *n*_WT_ = 6, *n*_*CosmcΔLEC*_ = 4 (E, F)). Data are given as geometric mean fluorescence intensity (gMFI) with each dot representing the result from one mouse. The columns represent the mean values ± SD. Unpaired Student's *t*‐test.

Strikingly, we observed increased immunofluorescence of F4/80 in the LN medullary sinus of mice with postnatal loss of *Cosmc* (Figure 4A) despite the reduced numbers of F4/80^+^ MSM detected by flow cytometry. This led us to quantify the expression levels of F4/80 as well as CD11b, Siglec‐1, SIGNR1, and the fractalkine receptor (CX3CR1) per single macrophage by flow cytometry (Figure 4B–F). We found increased levels of F4/80 protein on MSM, but not MCM (Figure 4D). Siglec‐1 expression on MSM and SSM of *Cosmc* ^*ΔLEC*^ mice was reduced, whereas SIGNR1 expression by MSM was not altered (Figure 4C,E). MSM but not SSM also displayed increased expression of CD80 and Ki‐67, suggesting increased immune activation and proliferation of the MSM (Figure S6A,B). Furthermore, the MCM in *Cosmc* ^*ΔLEC*^ mice displayed markedly increased expression of the fractalkine receptor CX3CR1 (Figure 4F).

Taken together, these results demonstrate significant changes in the sizes and phenotypes of the LN macrophage populations upon postnatal *Cosmc* deletion and loss of elongated core 1 O‐glycans. The total number of CD11b + macrophages and the number of MCM increased, and the latter expressed higher levels of CX3CR1; the sinusoidal macrophages SSM and MSM were reduced in numbers and in addition expressed lower levels of Siglec‐1. The reduced numbers of MSM were accompanied by increased cellular expression of F4/80 and immune activation markers, but not of SIGNR‐1.

## 4 Discussion

Our study has defined key functional roles for sialylated O‐glycans in shaping the molecular composition of the cell surface of lymphatic endothelia and in the formation of specific lymph node macrophage populations. Our analysis of the glycans present on cultured LEC derived from the LN of mice revealed that the disialylated core 1 O‐glycan is highly abundant (Figure 1B). The majority of N‐glycans and GSL are however non‐sialylated (Figure S1D,G). Interestingly, the N‐glycans present on the fLEC of the LN subcapsular sinus contain 3‐O‐sulfated galactose (Figure S1E,F), which is in agreement with our previous observation of sialidase‐resistant staining of fLEC by MAL‐1, which can bind 3‐O‐sulfated as well as α2,3‐sialylated glycans [9]. Previously, we had analyzed the O‐ and N‐glycomes of cultured, primary human dermal LEC. Interestingly, the O‐glycans were similar to those of the mouse LN LEC, but the N‐glycans differed substantially; in human dermal LEC, the N‐glycans were mostly complex and heavily sialylated, whereas on mouse LN LEC, N‐glycans of the oligomannose type predominated. To study the functional roles of sialylated O‐glycans in mouse LEC, we took two approaches to interfere with their biosynthesis, deleting either the sialyltransferase *St3Gal‐1* which encodes the α2–3 sialyltransferase that can sialylate core 1 O‐glycans, or deleting *Cosmc (C1Galt1C1)*, which encodes the chaperone that is essential for the function of T‐synthase which generates the core 1 O‐glycan.

T‐synthase is the only enzyme that adds galactose to the initial GalNAc (Tn antigen) of O‐glycans (Figure 1A). When inducing a functional loss of T‐synthase by deleting *Cosmc*, we observed that LN LEC lost their ability to synthesize sialylated core 1 O‐glycans and produced the sialyl Tn antigen instead (Figure 1B). In keeping with the results of the mass spectrometric analysis, cultured LEC derived from mouse LN and LEC in the subcapsular sinus of mouse LN were no longer stained by MAL‐II but were stained by HPA, consistent with the forced expression of the Tn antigen (Figure 1C–F). This observation confirms that the uniquely intense MAL‐II staining of the LN subcapsular sinus previously reported by us depended primarily on sialylated O‐glycans [9]. These findings led us to pursue our study here, using targeted deletion of Cosmc as the more effective means to eliminate α2,3‐sialylated O‐glycans from mouse LEC. It should be noted that mutations in *Cosmc* also occur in human diseases such as cancer, IgA nephropathy, Tn syndrome, and the multisystem chaperonopathies caused by germline mutations of *Cosmc* [35, 36, 37, 38]. Another key feature and outcome of our studies was identification of the role of specific sialyltransferases in sialylation of O‐glycans. St3Gal‐1 and St3Gal‐2 are two of the six known sialyltransferases that can add sialic acid in α2,3‐linkage to glycans, but until our study it was believed that St3Gal‐1 rather than St3Gal‐2 is primarily responsible for generating sialylated O‐glycans [29, 39]. Although deletion of St3Gal‐1 in LEC resulted in various alterations of the O‐glycan profile and an increase in non‐sialylated core 2 O‐glycan, representing the major structure (Figure S2B), we observed significant expression of mono‐ and di‐sialylated core 1 O‐glycans. This suggests that other sialyltransferases, likely St3Gal‐2, can also generate α2,3‐sialylated core 1 O‐glycans. Such studies as ours on deletions of specific sialyltransferases can help to define their in vivo specificities for glycosylation.

Interestingly, a key finding from our study is that the LEC from Cosmc^ΔLEC^ mice displayed strongly reduced abundances of several key O‐glycosylated glycoproteins, including podoplanin, Lyve‐1, and CD44 (Figure 2). Such changes were not observed for the LEC from *St3Gal‐1* ^*ΔLEC*^ mice (Figure S2G–M). Podoplanin, Lyve‐1, and CD44 are predicted to be densely O‐glycosylated, particularly in their membrane‐proximal parts (Net‐O‐Glyc; [40]). In contrast, the adhesion molecules ICAM‐1, VCAM‐1, and PECAM‐1/CD31, which are known to be heavily N‐glycosylated, were slightly increased in their abundances. None of these changes in glycoprotein abundance were paralleled by changes in the pertaining transcript levels (Figure S3A–F).

This latter observation is in line with the recent insight that regulatory processes occurring after gene transcription—post‐transcriptional, translational, and post‐translational processes, including the regulation of protein degradation—are of greater importance in controlling protein abundances under steady state conditions [41]. In addition to the synthesis of ligands for protein interactions, O‐glycosylation has at least two other key contributions to glycoprotein structures; it can be stabilizing in terms of overall protein structure, oligomerization, etc. [42, 43, 44], whereas O‐glycans located close to proteolytic cleavage sites and clustered O‐glycans near the cell membrane can regulate proteolysis and protein ectodomain shedding at the substrate level [31]. We found such a mechanism at play in the loss of CD44 from Cosmc^ΔLEC^ LEC, as they displayed large amounts of C‐terminal fragments of CD44 but had mostly lost the full‐length glycoprotein (Figure 2L). Studies on human CD44 have revealed that such 20–25 kDa C‐terminal fragments are generated by ectodomain cleavage mediated by the metalloprotease ADAM 10 [32, 34]. Previous reports on the regulation of proteolysis by O‐glycans were based on comparisons between ectodomain shedding of glycoproteins that had or had not been modified with O‐GalNAc by specific polypeptide GalNAc transferases (ppGalNTs; Figure 1A; [44]). By contrast, in the present study, the reductions in protein abundances occurred in glycoproteins modified with O‐GalNAc (i.e., the Tn antigen) but lacking extended O‐glycans. Therefore, not only the first O‐GalNAc attached by polypeptide GalNAc transferases, but also addition of galactose by T‐synthase appears to be important in posttranslational regulation of protein abundances and half‐lives. As T‐synthase is a unique enzyme whose function cannot be compensated for by other Gal transferases, the latter may be an even more efficient fine regulating factor.

Alterations in protein abundance have been reported in other studies where *Cosmc* was deleted in specific cell types. In platelets, loss of Cosmc/T‐synthase functions resulted in reduced abundance, impaired function, and partial proteolysis of the platelet glycoproteins GPIbα, GPVI, integrin αIIb and β3 [45]. In kidney podocytes, loss of Cosmc/T‐synthase functions resulted in loss of podoplanin, podocin, and nephrin [46]. In Cosmc‐deficient B‐cells somewhat higher abundances of L‐selectin and the integrin α4β7 were observed [47]. This latter observation is in line with our own finding of slightly increased abundances of the Ig‐like adhesion molecules ICAM‐1 and VCAM‐1 in LEC. One can only speculate on the underlying mechanisms. It could be that the increases occur to compensate for some loss in adhesivity of the LEC caused by the loss of podoplanin and the two hyaluronan receptors Lyve‐1 and CD44. Alternatively, one could imagine that due to a reduction of total glycoprotein content on the cell surface, the shedding of the remaining glycoproteins is reduced which results in their prolonged half‐lives. Taken together, loss of T‐synthase activity and the consequent loss of sialylated core 1‐derived O‐glycans on LEC leads to a fundamentally altered glycocalyx characterized by profound changes in the relative abundances of prominent cell membrane‐bound glycoproteins.

The altered glycocalyx of LEC would be expected to result in altered cell–cell interactions based on changes in the recognition by GBP, adhesion molecules and signaling receptors on other cells; immune cells are well equipped to sense patterns of glycans on invading pathogens and host cells.

Indeed, we observed that loss of Cosmc and functional T‐synthase in LEC dramatically influences the formation of LN macrophage populations in mice. When deletion of *Cosmc* was induced in newly born mice rather than in adult mice, the sizes and phenotypes of all lymph node macrophage populations investigated changed dramatically (Figure 3). At the age of 8 weeks, the *Cosmc* ^*ΔLEC*^ mice displayed an increase in the total number of LN macrophages along with a 2‐fold increase in the number of MCMs compared to WT mice (Figure 3B,C); expression of the fractalkine receptor CX3CR1 doubled in MCM (Figure 4F). In contrast, the SSM and MSM numbers were reduced by 40%–60% (Figure 3D,E). No other changes in the LN cellular composition or architecture were observed despite the presence of some red blood cells (Figure S5). This phenotype of blood‐filled lymphatics in LN of the neck region is caused by some retrograde filling from the blood circulation into the thoracic duct. It is also observed upon postnatal deletion of podoplanin and the reduced podoplanin levels in LEC of *Cosmc* ^*ΔLEC*^ mice are the likely cause [21]. Even though the phenotype of bloody lymph nodes was more severe in the *Pdpn* ^ΔLEC^ mice, it did not affect the integrity of high endothelial venules or the total number of leukocytes in the LN.

Both, SSM and MSM of *Cosmc* ^*ΔLEC*^ mice expressed lower levels of Siglec‐1 (Figure 4A,C). It is intriguing to note that loss of Siglec‐1 ligands (α2,3‐sialylated O‐glycans) on LEC was accompanied by reduced Siglec‐1 levels on sinusoidal macrophages; MSM additionally presented with higher levels of F4/80, but SIGNR‐1 expression remained the same (Figure 4D,E). It could be speculated that the unchanged SIGNR‐1 expression parallels the abundance of the oligomannose N‐glycans on LEC of *Cosmc* ^*ΔLEC*^ mice. But whether parallel changes in abundance of specific glycans and their presumably multiple cognate GBP on macrophages is a general pattern should be explored further. The MSM of *Cosmc* ^*ΔLEC*^ mice were furthermore more activated (increase in CD80 expression) and proliferative (increased Ki‐67 expression) compared to macrophages of WT controls (Figure S6).

We have previously observed more restricted changes in the numbers and/or phenotype of SSM and MSM in mice bearing a constitutional loss‐of‐function mutation in the carbohydrate binding domain of Siglec‐1 (*Siglec‐1* ^*W2QR97A*^ mice) [9]. Whereas there was only a trend toward increased MCM numbers and only the numbers of SSM were reduced, both SSM and MSM displayed a more activated and proliferative phenotype. In *Cosmc* ^*ΔLEC*^ mice, not only the Siglec‐1 ligands are reduced on the sinusoidal LEC, but also other components of a broader glycan‐dependent cellular niche are altered, including the glycoprotein composition of the cell surface and most probably also the release of signaling molecules.

Colony stimulating factor‐1 (CSF‐1) is a signaling molecule produced by sinusoidal LN LEC that has been found to support homing, differentiation, and immune functions of sinusoidal macrophages [19]. It is interesting to note that CSF‐1, in addition to three N‐glycans in its N‐terminal part, is predicted to display abundant O‐glycans (NetOGlyc4; [40]). CSF‐1 occurs as a membrane bound glycoprotein whose ectodomain can be shed by proteases such as ADAM17 [48, 49]. Protein shedding by ADAM17 can be coregulated by O‐glycosylation and may thus occur more readily in the LEC of *Cosmc* ^*ΔLEC*^ mice [50]. The resulting changes in the relative abundances of membrane‐bound and soluble CSF‐1 and the pertaining effects on macrophage development may underly the impaired formation of both the SSM and MSM networks. Our observation of a concomitant increase in MCM numbers may support the hypothesis that the sinusoidal macrophages develop from MCM [10]. With possible changes in the release of signaling mediators such as CSF‐1 upon loss of sialylated O‐glycans by sinusoidal LEC, development of SSM and MSM from MCM may be impaired and MCM accumulate in the LN medulla.

That deletion of Cosmc altered the size and phenotypes of LN macrophage populations only when induced postnatally, suggests that proper O‐glycosylation in LEC is important in the early time window when the sinusoidal macrophage populations are established from a postnatal wave of BM‐derived monocytes immigrating into the LN [19]. Macrophage differentiation and proliferation then depend on the local availability of chemotactic and survival factors such as lymphotoxin (LT) α1𝛽1 produced by B cells, RANK ligand expressed by marginal reticular cells, as well as CSF‐1 produced by LEC [12, 18, 19].

In summary, we show that the loss of T‐synthase function induced by deletion of Cosmc in LEC has profound implications for the glycocalyx of these cells. Not only did they lose their sialylated core 1 O‐glycans, but they also underwent major alterations in the relative abundances of O‐glycoproteins and, to a lesser extent, Ig‐like adhesion molecules. These changes in molecular composition are probably based on multiple, only partially understood mechanisms that are likely to include enhanced proteolysis and ectodomain shedding of O‐glycoproteins displaying the Tn antigen and some unknown compensatory mechanisms that increase the abundance of Ig‐like adhesion molecules. The altered LEC glycocalyx translated into multiple changes in the cellular niche for LN macrophages that presumably include altered levels and sensing of signaling mediators and altered cell–cell interactions mediated by the multiple macrophage GBP such as Siglec‐1 and other adhesion molecules. Loss‐of‐function of T‐synthase on LEC shortly after birth led to profound alterations in the macrophage populations in the mouse lymph node regarding both their sizes and their phenotypes. These findings imply that T‐synthase activity is a major regulating valve for the molecular composition of the LEC cell surface and secretome and the consequent interactions with monocytes/macrophages. It has its major impact in the first weeks after birth when the monocytes immigrate into the lymph node and differentiate into the specialized macrophage populations that reside in the lymphatic sinus and medullary cords.

## Author Contributions

Jasmin Frey designed and performed experiments, analyzed and interpreted data and drafted the manuscript. Annkathrin Ratter designed and performed the Western blot analyses of LN LEC lysates and contributed to the revision of the manuscript. Finn Brigger synthesized the CORA reporter, isolated and purified Bn‐*O‐*GalNAc glycans. Louie Cielen, Pascal Kunz, and Ea Tulin established and performed the immunofluorescent stainings of glycoproteins, and Kinga Mikos performed the RT‐PCR measurements. Lothar C. Dieterich and Marco D'Addio provided valuable intellectual input on experimental procedures and data analysis. Cornelia Halin provided valuable material and intellectual input on mouse experiments. Richard D. Cummings gave valuable intellectual input on T‐synthase biology, the observed changes in the glycoproteome, and thoroughly revised the manuscript. Vivianne I. Otto designed, planned, and supervised the study and wrote the manuscript.

## Funding

This work was supported by OPO‐Stiftung (OPO‐Foundation) (2018‐0048, 2020‐0018). Novartis Stiftung für Medizinisch‐Biologische Forschung (Novartis Foundation for Medical‐Biological Research) (22C‐234). OPO‐Stiftung (OPO‐Foundation) (2025‐0009). Vontobel‐Stiftung (Vontobel Foundation) (0264/2024).

## Conflicts of Interest

The authors declare no conflicts of interest.

## Supporting information


**Figure S1:** fsb272282‐sup‐0001‐FigureS1.pdf.

**Figure S2:** fsb272282‐sup‐0002‐FigureS2.pdf.

**Figure S3:** fsb272282‐sup‐0003‐FigureS3.pdf.

**Figure S4:** fsb272282‐sup‐0004‐FigureS4.pdf.

**Figure S5:** fsb272282‐sup‐0005‐FigureS5.pdf.

**Figure S6:** fsb272282‐sup‐0006‐FigureS6.pdf.

## Data Availability

The data that support the findings of this study will be openly available in the ETH Research Collection at https://doi.org/10.3929/ethz‐c‐000792736 upon publication of the manuscript.
