# Supplementary material for: Protein O‐Glycosylation Shapes Lymphatic Endothelia and Lymph Node Macrophage Populations
Source: FASEB J. 2026 Sep 11;40(18):e72282. doi: 10.1096/fj.202600240R (PMC13565275; doi:10.1096/fj.202600240R)
Supplement: Supplementary file 1 [file FSB2-40-e72282-s001.pdf]

Supplemental Figure 4

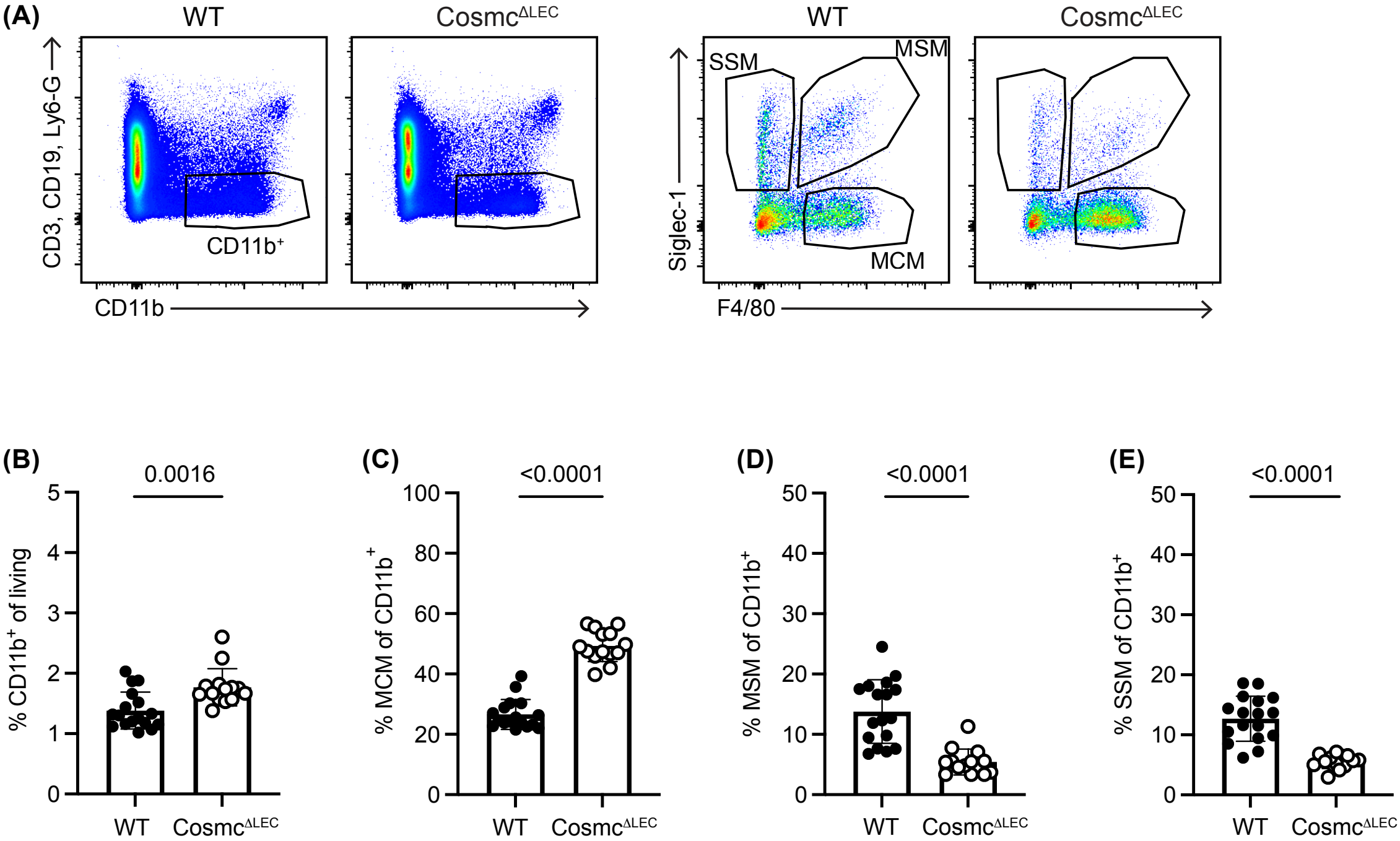

Supplemental Figure 4: *Gating strategy for quantification of the macrophage populations in the mouse lymph node.* **(A)** The gating strategy is shown using representative flow cytometric plots of auricular LNs isolated from mice treated with tamoxifen postnatally (experimental set-up shown in **Figure 3A**) gated for single live cells. $CD3^{+}$ $CD19^{+}$ lymphocytes and LY6-G^{+} neutrophils were excluded ($dump^{+}$ cells; flow cytometric plots on the left). The three LN macrophage subpopulations were gated from the $dump^{-}$ $CD11b^{+}$ cells based on their expression of Siglec-1 and F4/80: Subcapsular sinus macrophages (SSM: Siglec-1^{+} $F4/80^{-}$), medullary sinus macrophages (MSM: Siglec-1^{+} $F4/80^{+}$), medullary cord macrophages (MCM: Siglec-1^{-} $F4/80^{+}$). The percentage of CD11b+ among the living single cells **(B)** and the percentages of MCM **(C)**, MSM **(D)**, and SSM **(E)** among the cCD11b+ LN macrophages are shown. The data derive from 3 pooled experiments (n_{WT} = 17, $n_{Cosmc\Delta LEC}$ =14) and the single as well as the mean values +/- SD are shown. Unpaired Student's t-test.
