# Supplementary material for: Protein O‐Glycosylation Shapes Lymphatic Endothelia and Lymph Node Macrophage Populations
Source: FASEB J. 2026 Sep 11;40(18):e72282. doi: 10.1096/fj.202600240R (PMC13565275; doi:10.1096/fj.202600240R)
Supplement: Supplementary file 2 [file FSB2-40-e72282-s002.pdf]

Supplemental Figure 3

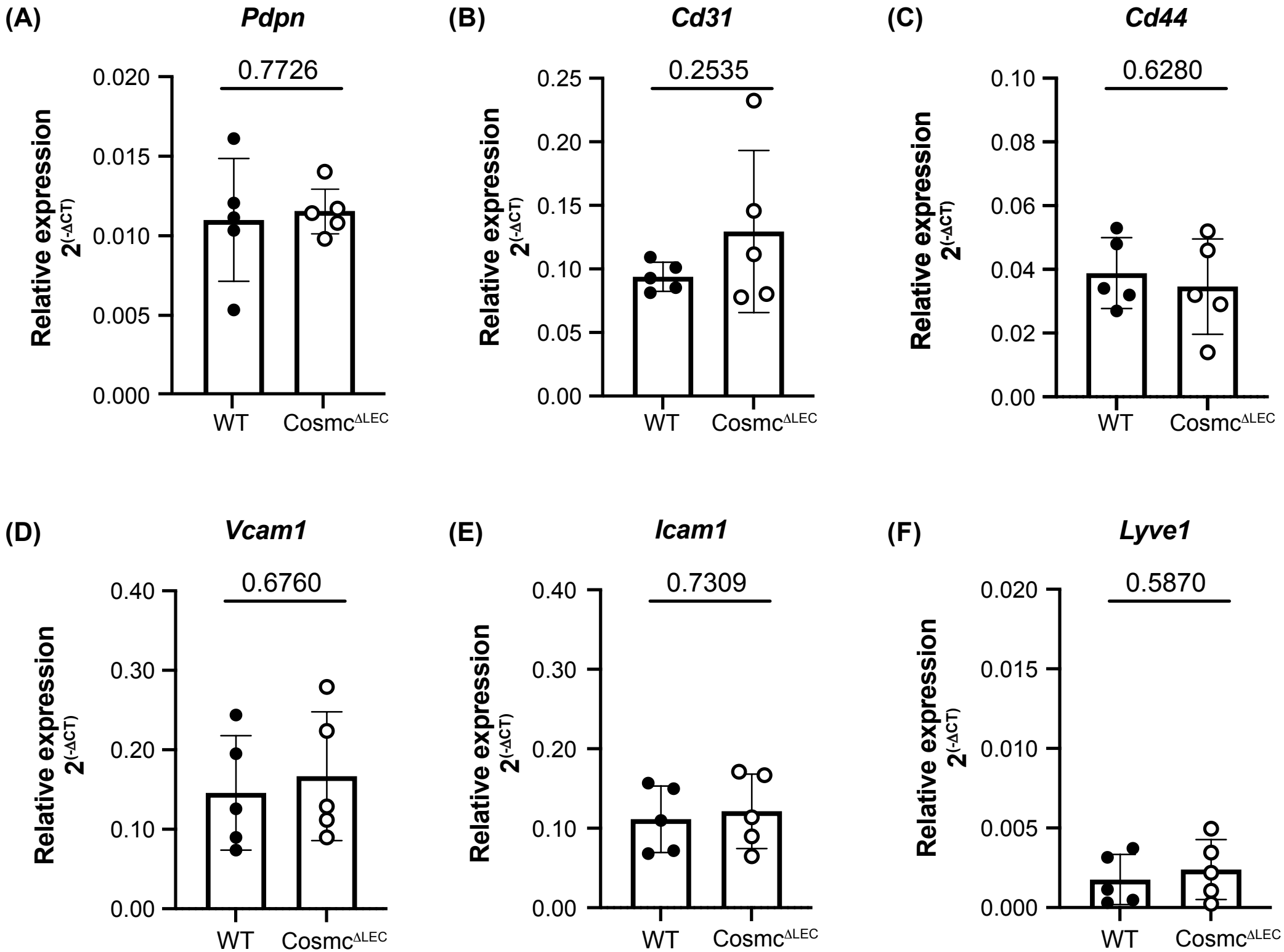

Supplemental Figure 3: *The altered abundances of prominent glycoproteins upon loss of Cosmc/T-synthase function in lymphatic endothelial cells are not paralleled by the pertaining transcript levels.* Cultured LN LEC that were derived from WT and *Cosmc*$^{\Delta LEC}$ were analyzed by qPCR for the transcript levels of **(A)** podoplanin /*Pdpn*, **(B)** *Cd31*, **(C)** *Cd44*, **(D)** *Vcam1*, (**E**) *Icam1*, and (**F**) *Lyve1*. Shown are relative expression levels normalized to the expression of the house keeping gene Gapdh. Unpaired Student's t-test.
