# Supplementary material for: Protein O‐Glycosylation Shapes Lymphatic Endothelia and Lymph Node Macrophage Populations
Source: FASEB J. 2026 Sep 11;40(18):e72282. doi: 10.1096/fj.202600240R (PMC13565275; doi:10.1096/fj.202600240R)
Supplement: Supplementary file 3 [file FSB2-40-e72282-s003.pdf]

Supplemental Figure 2

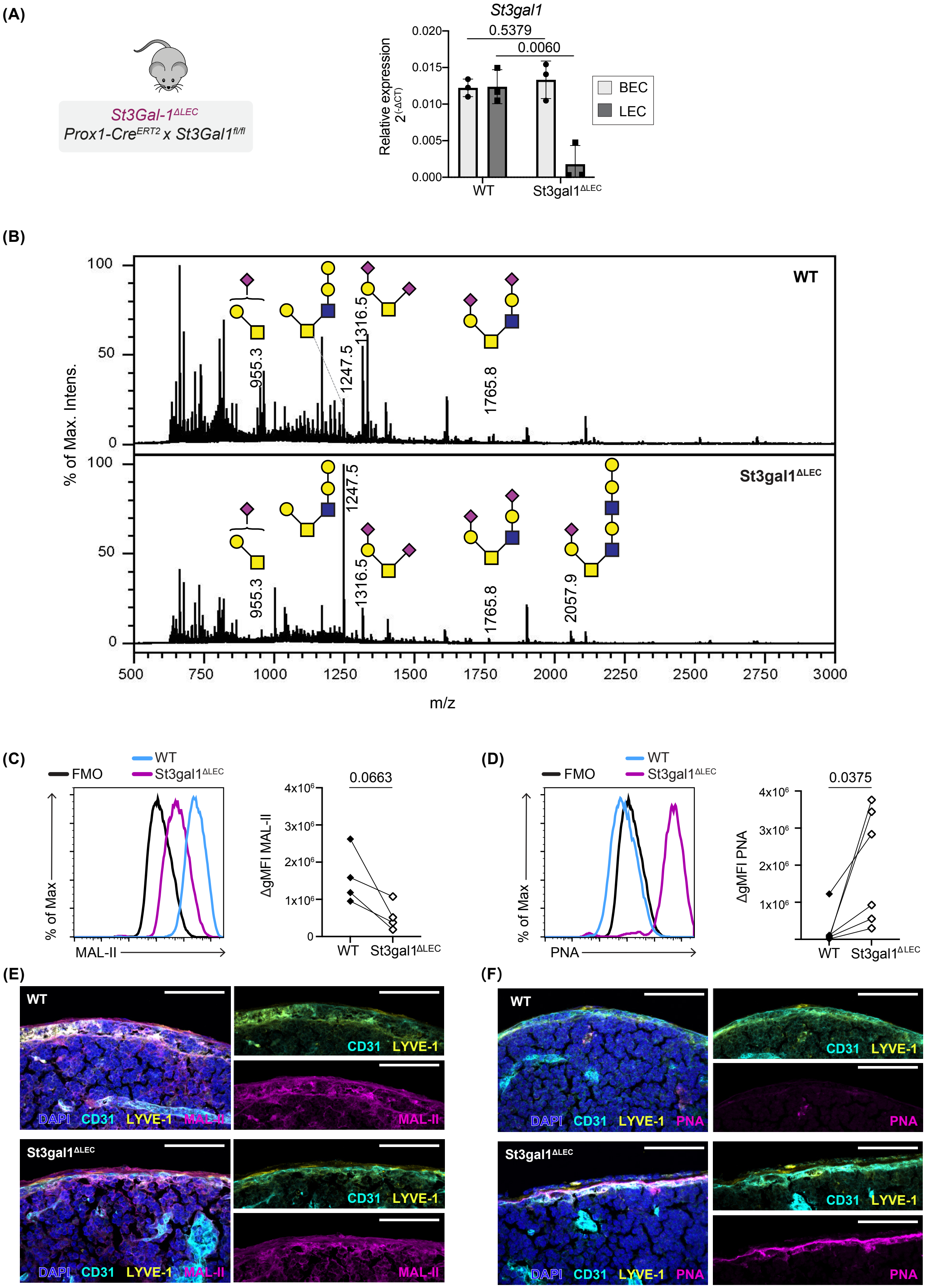

(G)

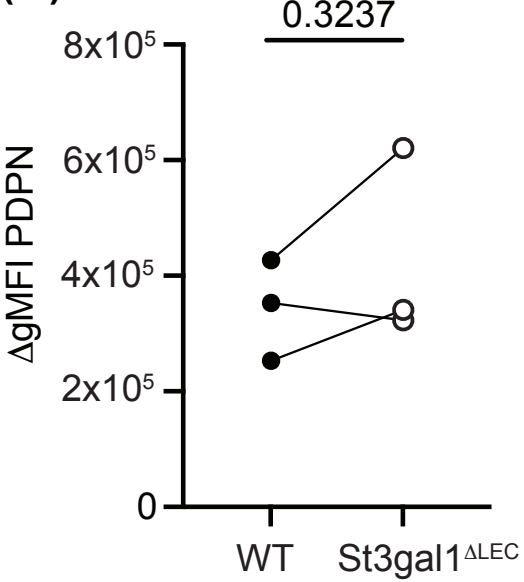
0.3237
ΔgMFI PDPN
8x10⁵
6x10⁵
4x10⁵
2x10⁵
0
WT
St3gal1ΔLEC

(H)

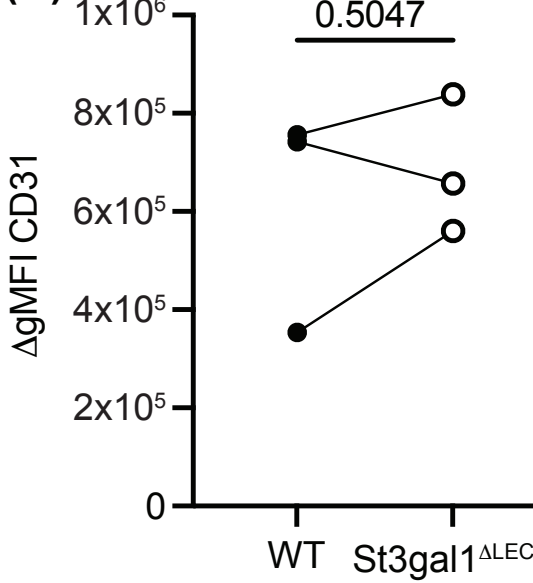
0.5047
ΔgMFI CD31
1x10⁶
8x10⁵
6x10⁵
4x10⁵
2x10⁵
0
WT
St3gal1ΔLEC

(I)

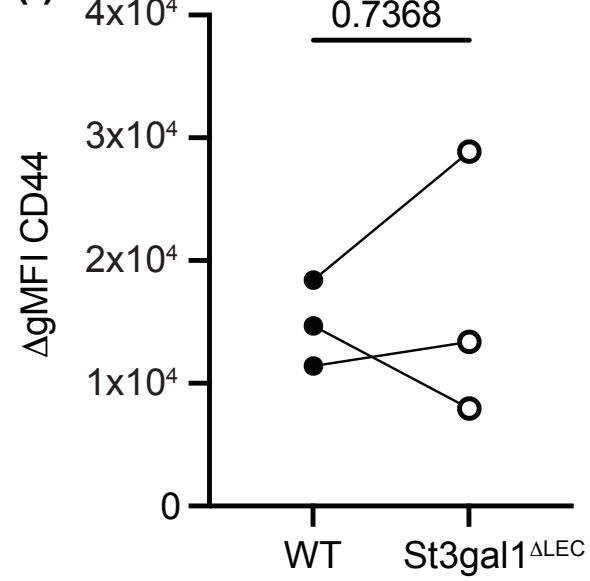
0.7368
ΔgMFI CD44
4x10⁴
3x10⁴
2x10⁴
1x10⁴
0
WT
St3gal1ΔLEC

(K)

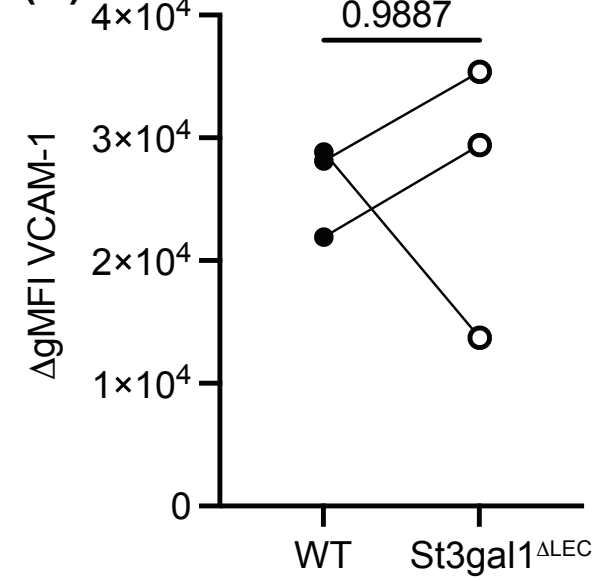
0.9887
ΔgMFI VCAM-1
4×10⁴
3×10⁴
2×10⁴
1×10⁴
0
WT
St3gal1ΔLEC

(L)

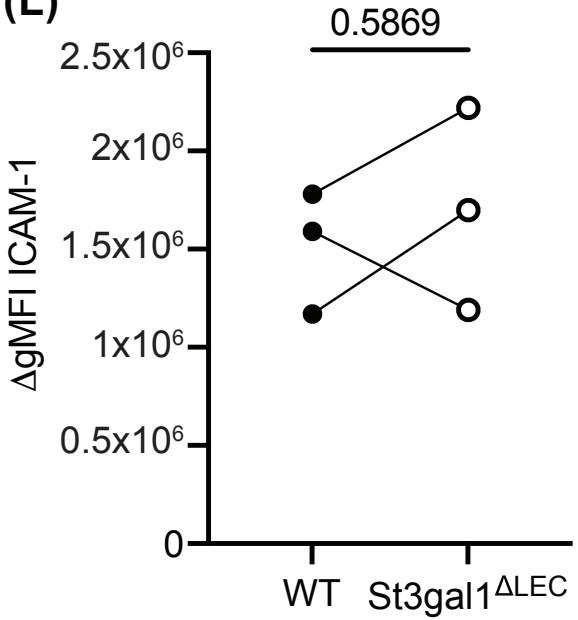
0.5869
ΔgMFI ICAM-1
2.5x10⁶
2x10⁶
1.5x10⁶
1x10⁶
0.5x10⁶
0
WT
St3gal1ΔLEC

(M)

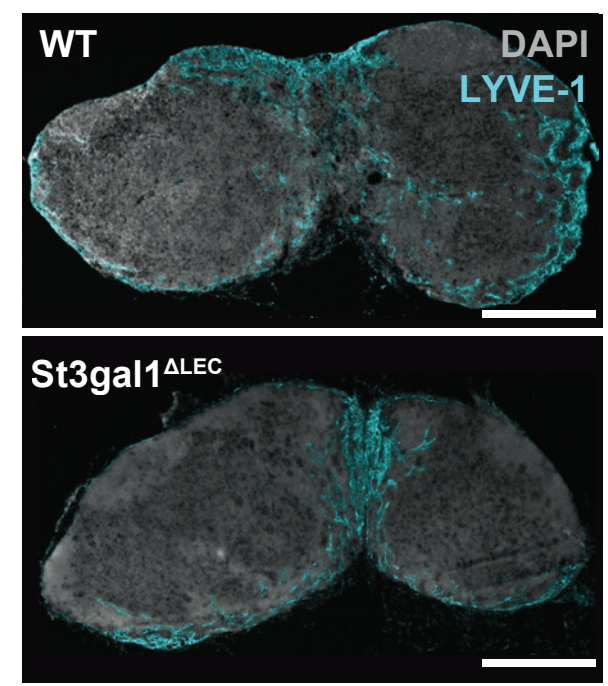
WT
DAPI
LYVE-1
St3gal1ΔLEC

Supplemental Figure 2:

Deletion of St3gal1 does not abolish the synthesis of α2,3-sialylated O-glycans by lymphatic endothelial cells.

(**A**) The approach used to generate an inducible, lymphatic endothelial cell (LEC)-specific deletion of St3gal1. Upon treatment of mice with tamoxifen, Cre^{ERT2} recombinase deletes the floxed alleles of *St3Gal1* in PROX1-expressing cells. Mice were treated with tamoxifen postnatally (P1-P3) and lymph nodes isolated when they were 8 weeks-old. The relative expression of *St3gal1* was assessed by qPCR in LEC and BEC sorted by flow cytometry from lymph node (LN) single cell suspensions in *St3gal1*$^{\Delta LEC}$ mice and their WT littermates (n=3).

(**B**) MALDI-TOF MS of permethylated Bn-O-glycans produced by cultured, primary LN LEC upon incubation with the CORA reporter (Ac_3GalNAc-α-Bn). The cultured primary LN LEC had been isolated from WT and *St3gal1*$^{\Delta LEC}$ littermates. Peaks are scaled relative to the maximum signal intensity. Putative glycan structures are shown next to the corresponding peaks. Unlabelled peaks derive from non-carbohydrate impurities.

(**C, D**) MAL-II (*Maackia amurensis* lectin-II) and PNA (peanut agglutinin) binding to cultured, primary LN LEC that had been isolated from WT and *St3gal1*$^{\Delta LEC}$ mice. LN LEC stained omitting the lectin were used as negative FMO (fluorescence minus one) controls. Representative flow cytometric histograms and summary of the delta geometric mean fluorescence intensities (ΔgMFI) of 4 or 6 independent experiments. Data derived from the same experiment are connected by a line. Paired Student's t-test.

(**E, F**) Binding of MAL-II and PNA to LN sections of eight-week-old WT and *St3gal1*$^{\Delta LEC}$ mice treated with tamoxifen postnatally, counter-stained for cell nuclei (DAPI), endothelial cells (CD31), and LEC (Lyve-1). Scale bars are 50 µm.

Cultured, primary LN LEC that had been isolated from WT and *St3gal1*$^{\Delta LEC}$ mice were analyzed by flow cytometry for the expression of (**G**) podoplanin /PDPN, (**H**) CD31, (**I**) CD44, (**K**) VCAM-1, (**L**) ICAM-1. The delta geometric mean fluorescence intensities (ΔgMFI) of 3 independent experiments are shown. Data deriving from the same experiment are connected by a line. Paired Student's t-test. (**M**) The LN of WT and *St3gal1*$^{\Delta LEC}$ were analyzed for the expression of Lyve-1 by immunofluorescence. Scale bars are 500 µm.
