# Supplementary material for: Protein O‐Glycosylation Shapes Lymphatic Endothelia and Lymph Node Macrophage Populations
Source: FASEB J. 2026 Sep 11;40(18):e72282. doi: 10.1096/fj.202600240R (PMC13565275; doi:10.1096/fj.202600240R)
Supplement: Supplementary file 4 [file FSB2-40-e72282-s004.pdf]

Supplemental Figure 1

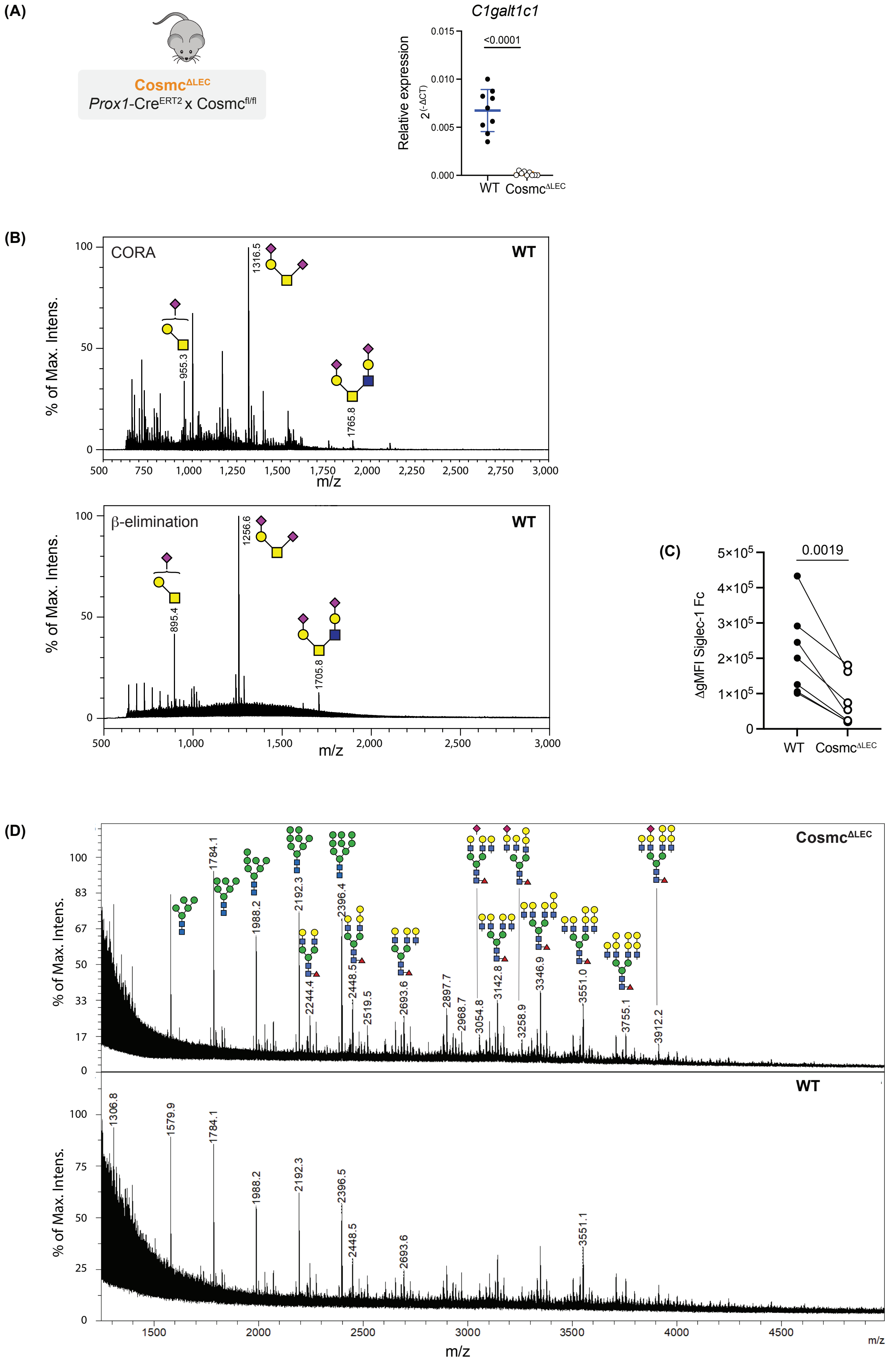

(E)

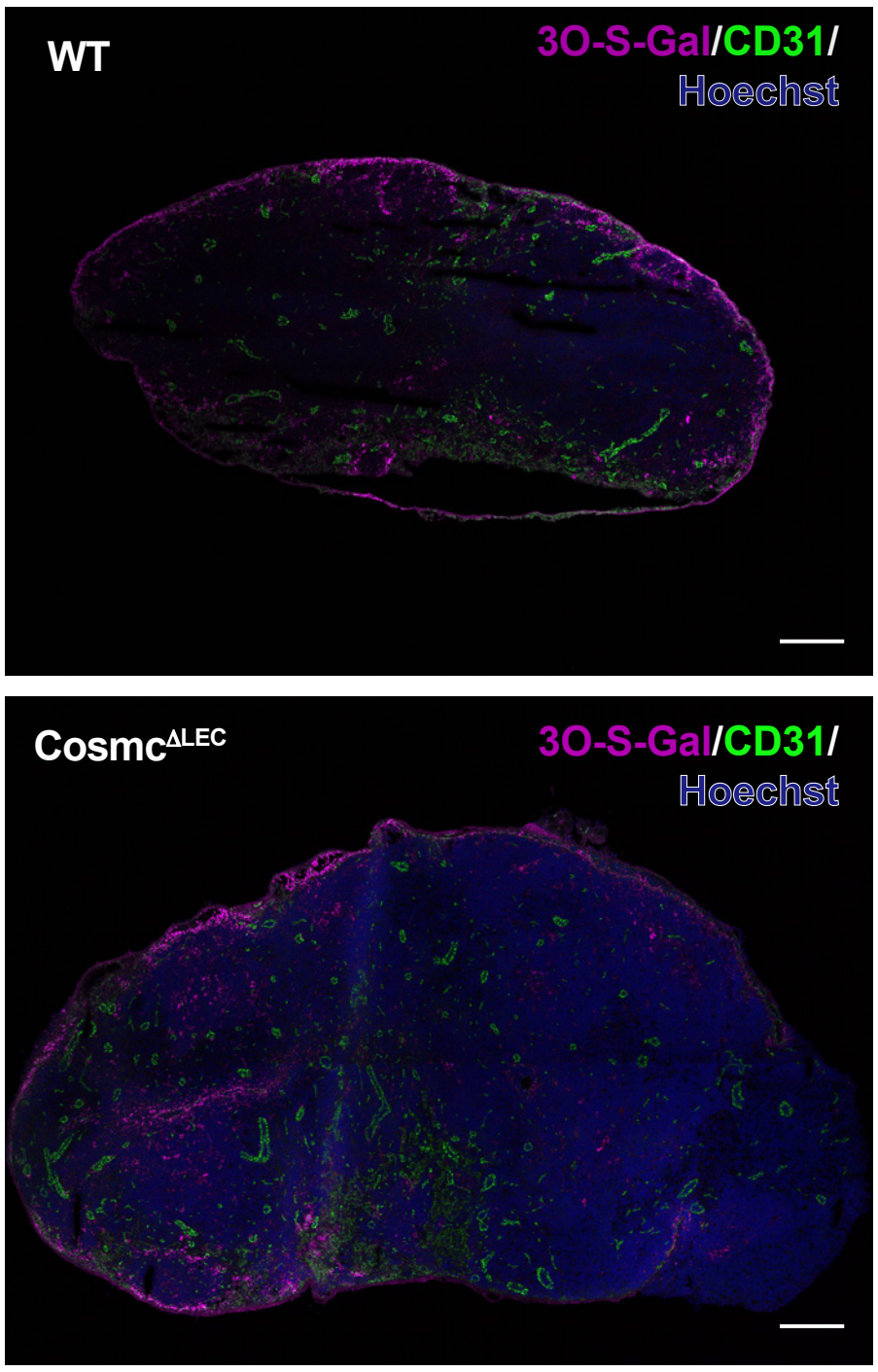

WT
3O-S-Gal/CD31/
Hoechst
Cosmc$^{\Delta LEC}$
3O-S-Gal/CD31/
Hoechst

(F)

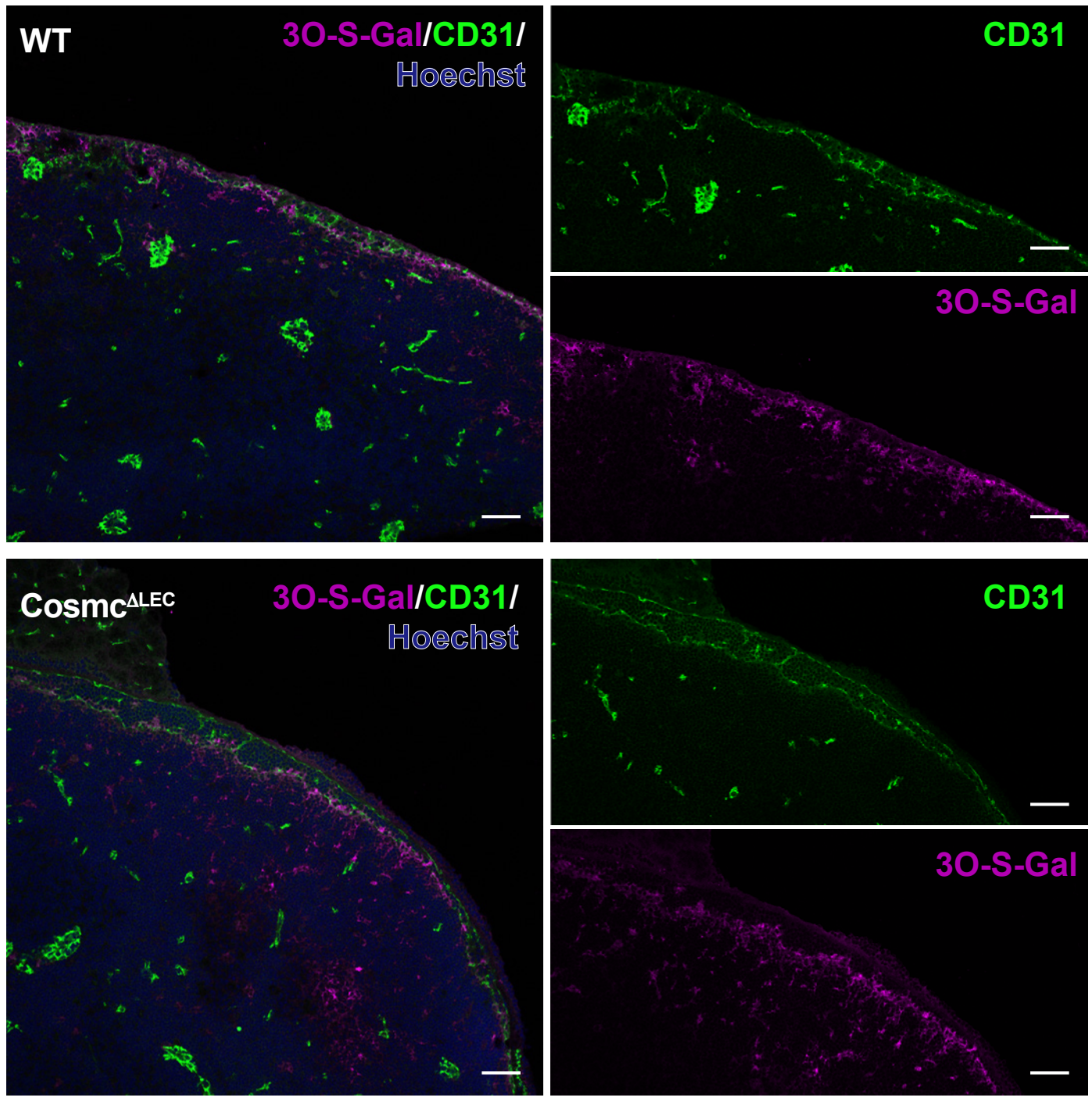

WT
3O-S-Gal/CD31/
Hoechst
CD31
3O-S-Gal
Cosmc$^{\Delta LEC}$
3O-S-Gal/CD31/
Hoechst
CD31
3O-S-Gal

(G)

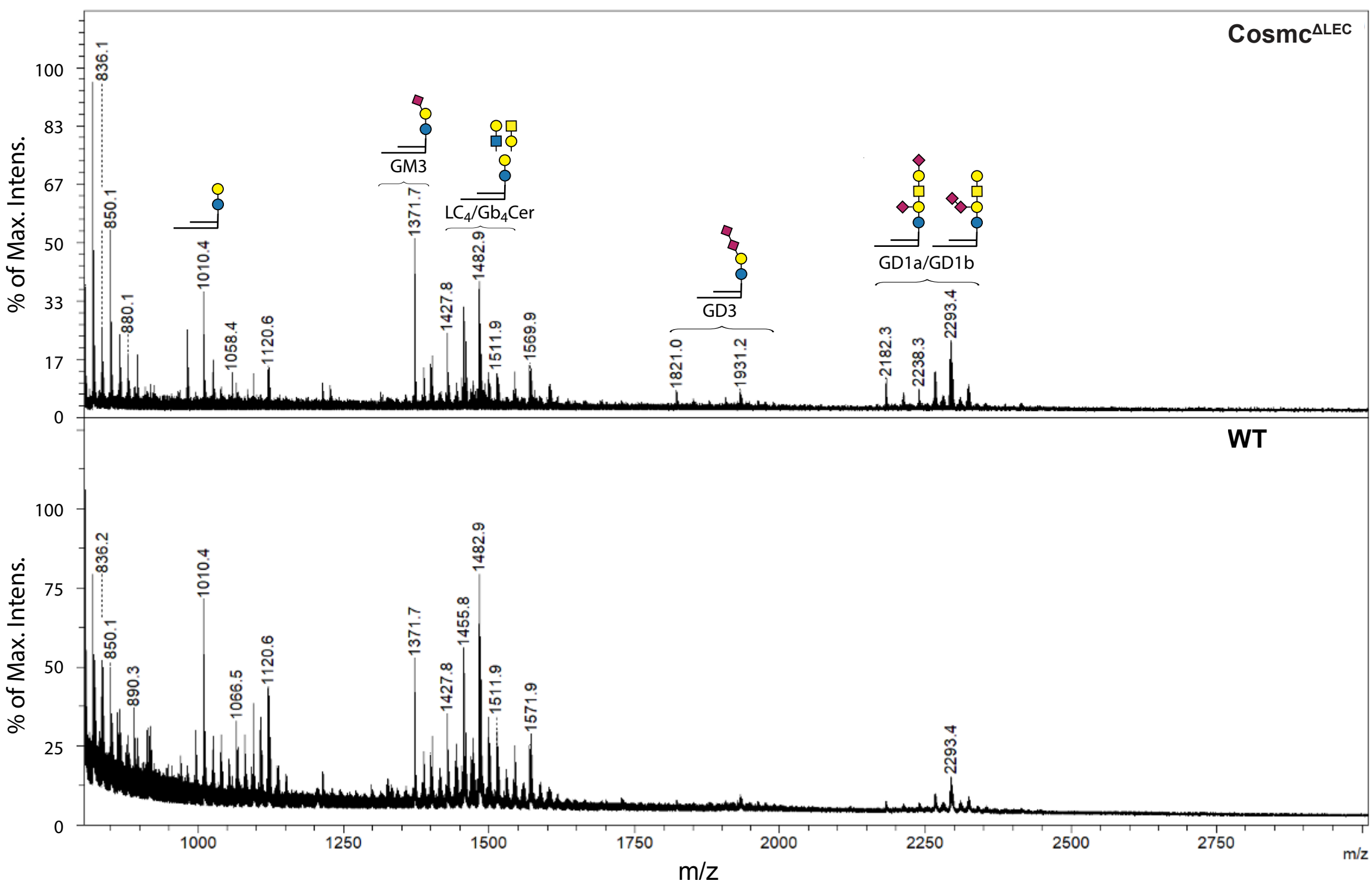

Cosmc$^{\Delta LEC}$
% of Max. Intens.
100
83
67
50
33
17
0
836.1
850.1
880.1
1010.4
1058.4
1120.6
GM3
1371.7
1427.8
LC$_4$/Gb$_4$Cer
1482.9
1511.9
1569.9
GD3
1821.0
1931.2
GD1a/GD1b
2182.3
2238.3
2293.4
WT
% of Max. Intens.
100
75
50
25
0
836.2
850.1
890.3
1010.4
1066.5
1120.6
1371.7
1427.8
1455.8
1482.9
1511.9
1571.9
2293.4
1000
1250
1500
1750
2000
2250
2500
2750
m/z
m/z

Supplemental Figure 1:

*Loss of Cosmc/T-synthase function causes loss of Siglec-1 ligands but does not affect the N-glycan or glycosphingolipid profiles of lymphatic endothelial cells***.** (**A**) The approach used to generate an inducible, lymphatic endothelial cell (LEC)-specific deletion of *Cosmc* (*C1galt1c1)*. Upon treatment of mice with tamoxifen, Cre^{ERT2} recombinase deletes the floxed allele(s) of *C1galt1c1* in PROX1-expressing cells. *C1galt1c1* transcripts in cultured, primary lymph node (LN) LEC from either wild-type (WT) or $Cosmc^{\Delta LEC}$ mice (n=9) were assessed by qRT-PCR. Unpaired student's t-test.

(**B**) MALDI-TOF MS of either permethylated benzyl (Bn)-O-glycans secreted by cultured, primary LN LEC that have been incubated with the CORA reporter (Ac_3GalNAc-α-Bn; upper panel) or of permethylated O-glycans released from LN LEC by β-elimination (lower panel). The cultured, primary LN LEC had been isolated from WT mice. Peaks are scaled relative to the maximum signal intensity. Putative glycan structures matching the detected masses are shown next to the corresponding peak. Unlabeled peaks derive from non-carbohydrate impurities. The same cellular O-glycan profile was observed with both methods.

(**C**) Binding of recombinant Siglec-1-Fc to lymphatic endothelial cells (LEC) isolated from lymph nodes of either WT or $Cosmc^{\Delta LEC}$ mice was assessed by flow cytometry. The delta geometric mean fluorescent intensities (ΔgMFI) of 7 independent experiments are given. Student's paired t-test. (**D**) MALDI-TOF MS profile of permethylated *N*-glycans released from the LN LEC isolated from $Cosmc^{\Delta LEC}$ and WT mice. (**E, F**) LN sections from eight-week-old WT and $Cosmc^{\Delta LEC}$ mice that had been treated with tamoxifen postnatally were analyzed for glycans containing 3-O-sulfated galactose. CD31 was used for localization of LEC. Whole LN scans (**E**) and enlarged sections of LN focusing on the subcapsular sinus (**F**) are shown. Staining was strictly limited to the subcapsular sinus fLEC, with neither cLEC, LEC in the medullary sinus or high endothelial venules being stained. The unaltered staining on the fLEC in the LN of $Cosmc^{\Delta LEC}$ mice compared to WT mice shows that the 3-O-sulfated galactose is present on N-glycans and not on O-glycans. Scale bars are 200 µm (**E**) and 50 µm (**F**).

(**G**) MALDI-TOF MS profile of permethylated glycosphingolipids (GSL) extracted from LN LEC. Identification of individual GSL was based primarily on their determined molecular masses, facilitated by the knowledge of the structures of the glycan head groups of ganglio-, lacto-, and globo-series. The range for GSL with the same glycan moiety that is created by the typical heterogeneity of the fatty acyl groups of Ceramide (Cer) is depicted with curly brackets. Peaks are scaled relative to the maximum intensity. Unlabeled peaks derive from non-carbohydrate impurities.
