# Supplementary material for: Protein O‐Glycosylation Shapes Lymphatic Endothelia and Lymph Node Macrophage Populations
Source: FASEB J. 2026 Sep 11;40(18):e72282. doi: 10.1096/fj.202600240R (PMC13565275; doi:10.1096/fj.202600240R)
Supplement: Supplementary file 5 [file FSB2-40-e72282-s005.pdf]

Supplemental Figure 5

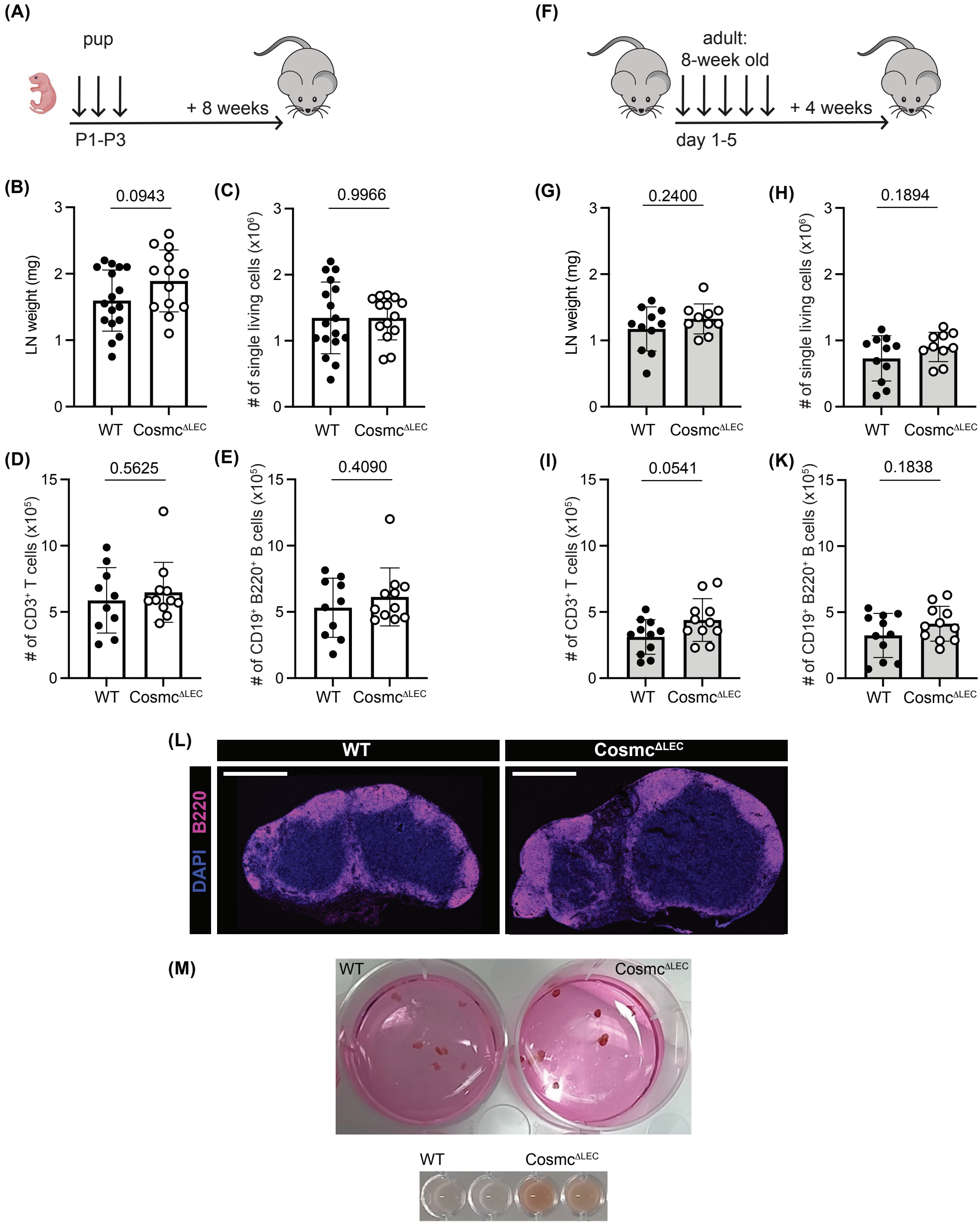

Supplemental Figure 5: *Loss of Cosmc/T synthase activity in lymphatic endothelial cells of either pups or adult mice does not alter the lymph node weight, architecture, cellularity, or B and T cell populations.* The auricular lymph nodes of mice in whom loss of T-synthase function was induced in LEC either postnatally (**A**) or at the age of 8 weeks (**F**) were analyzed for their weights (**B, G**; average of 2 auricular LNs per mouse), total cellularity (**C, H**; numbers (#) of single living cells), numbers of $CD3^{+}$ T cells (**D, I**; 1 dot = 1 auricular LN per mouse) and of $CD19^{+}$ $B220^{+}$ B cells (**E, K**; 1 dot = 1 auricular LN per mouse). Pooled data from 1-3 experiments measured by flow cytometry (total numbers of mice: in T and B cell analysis: $n_{Cosmc\ deleted\ postnatally}$ = 10 / 11; $n_{Cosmc^{-}\ deleted\ in\ adults}$ = 11 / 11, in analysis of LN weights and total cellularity: $n_{Cosmc\ deleted\ postnatally}$ = 17 / 13; $n_{Cosmc^{-}\ deleted\ in\ adults}$ = 11 / 10). Unpaired student's t-test. (**L**) Visualization of lymph node architecture by staining for cell nuclei (DAPI) and B cell follicles (B220) in auricular LN sections from eight-week-old WT mice and their *Cosmc*$^{\Delta LEC}$ littermates in whom Cosmc deletion had been induced postnatally. Scale bars are 500 µm.

After postnatal deletion of Cosmc in LEC, we frequently observed that the LN in adult *Cosmc*$^{\Delta LEC}$ mice were redder than in their WT littermates (**M**). Freshly isolated LN and LN single cell suspensions are shown. Together with the trend toward slightly increased weights of auricular LN observed in *Cosmc*$^{\Delta LEC}$ mice (**B**), this suggests the presence of red blood cells in the LN and thus spilling of blood into the lymphatic vessels. Mice with a constitutional loss of T-synthase in endothelial and hematopoietic cells have been reported to die during embryonic development, after birth or weaning, and exhibit disorganized, blood-filled lymphatics (1). The separation of blood and lymphatic vessels during embryonic development depends on endothelial O-glycans, expression of the heavily O-glycosylated podoplanin and its binding by CLEC-2. Similarly, deletion of podoplanin is lethal after birth and accompanied by blood-filled lymphatics and poor LN organization (2). Mice with LEC-specific postnatal deletion of podoplanin (*Pdpn*$^{\Delta LEC}$ mice) exhibited normal organization of lymphatic vessels and LN, but lymphatic vessels and LN were often filled with blood (3). The phenotype of blood-filled LN that we have observed in *Cosmc*$^{\Delta LEC}$ mice was milder than in *Pdpn*$^{\Delta LEC}$ mice, which may be explained by some residual expression of podoplanin in *Cosmc*$^{\Delta LEC}$ mice (**Figure 2A, H**). Bloody LN or increased LN weight were however not observed upon LEC-specific gene deletion of *Cosmc* or *Pdpn* in adult mice (**G**, (4)).

References

1. Fu, J., Gerhardt, H., McDaniel, J. M., Xia, B., Liu, X., Ivanciu, L., Ny, A., Hermans, K., Silasi-Mansat, R., McGee, S., Nye, E., Ju, T., Ramirez, M. I., Carmeliet, P., Cummings, R. D., Lupu, F., and Xia, L. (2008) Endothelial cell O-glycan deficiency causes blood/lymphatic misconnections and consequent fatty liver disease in mice. *The Journal of clinical investigation* **118**, 3725-3737
2. Uhrin, P., Zaujec, J., Breuss, J. M., Olcaydu, D., Chrenek, P., Stockinger, H., Fuertbauer, E., Moser, M., Haiko, P., Fassler, R., Alitalo, K., Binder, B. R., and Kerjaschki, D. (2010) Novel function for blood platelets and podoplanin in developmental separation of blood and lymphatic circulation. *Blood* **115**, 3997-4005
3. Bianchi, R., Russo, E., Bachmann, S. B., Proulx, S. T., Sesartic, M., Smaadahl, N., Watson, S. P., Buckley, C. D., Halin, C., and Detmar, M. (2017) Postnatal Deletion of Podoplanin in Lymphatic Endothelium

Results in Blood Filling of the Lymphatic System and Impairs Dendritic Cell Migration to Lymph Nodes. *Arterioscler Thromb Vasc Biol* **37**, 108-117

4. Sibler, E., He, Y., Ducoli, L., Rihs, V., Sidler, P., Puig-Moreno, C., Frey, J., Fujimoto, N., Detmar, M., and Dieterich, L. C. (2022) Immunomodulatory Responses of Subcapsular Sinus Floor Lymphatic Endothelial Cells in Tumor-Draining Lymph Nodes. *Cancers (Basel)* **14**
