# Supplementary material for: Protein O‐Glycosylation Shapes Lymphatic Endothelia and Lymph Node Macrophage Populations
Source: FASEB J. 2026 Sep 11;40(18):e72282. doi: 10.1096/fj.202600240R (PMC13565275; doi:10.1096/fj.202600240R)
Supplement: Supplementary file 6 [file FSB2-40-e72282-s006.pdf]

Supplemental Figure 6

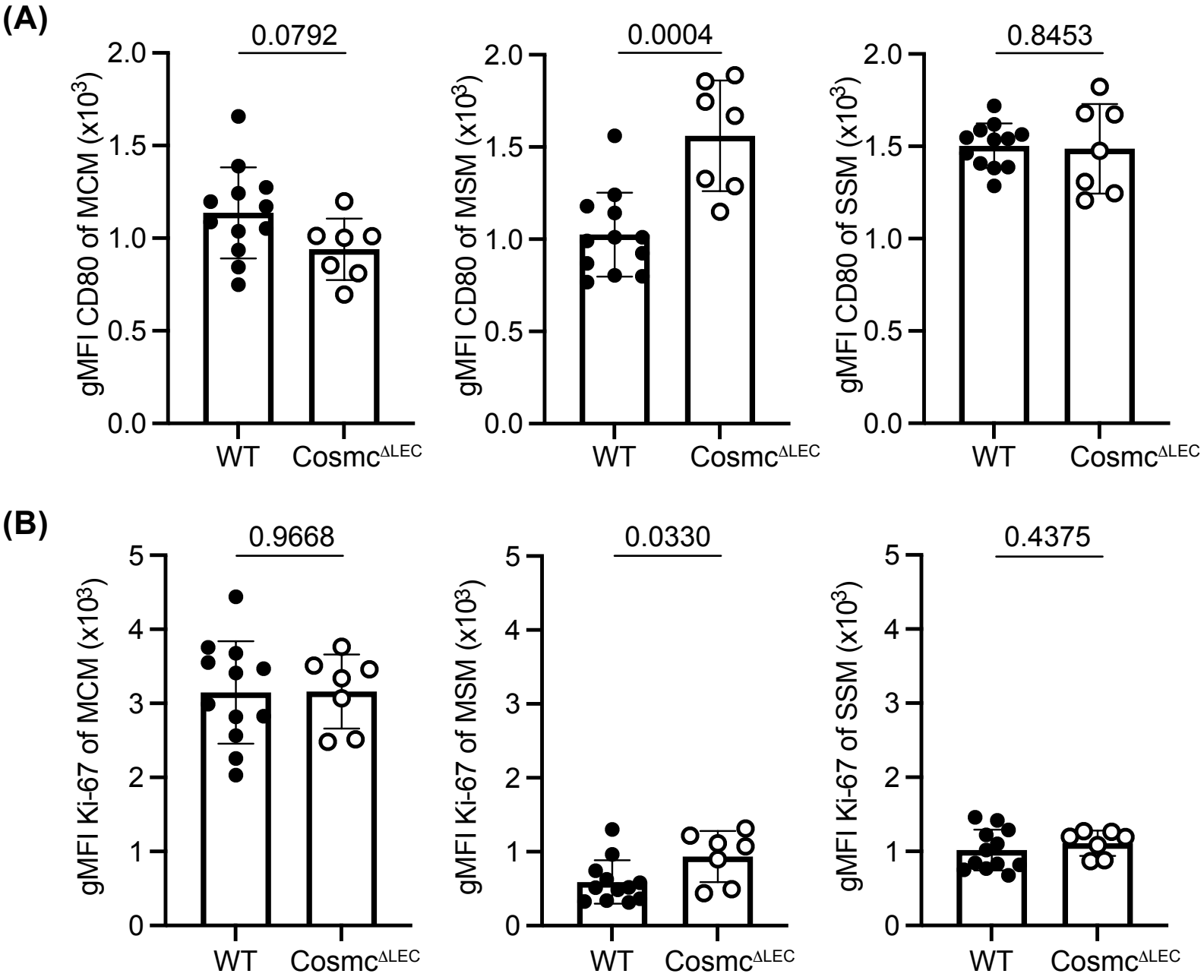

Supplemental Figure 6. *Loss of Cosmc/T-synthase activity in lymphatic endothelial cells after birth confers medullary sinus macrophages with a more activated, proinflammatory and proliferative phenotype.* Expression of the activation marker CD80 (**A**) and the proliferation marker Ki-67 (**B**) by medullary cord macrophages (MCM), medullary sinus macrophages (MSM) and subcapsular sinus macrophages (SSM) isolated from the auricular lymph nodes of adult wild type (WT) and $Cosmc^{\Delta LEC}$ mice that had been treated with tamoxifen postnatally was measured by flow cytometry (2-3 pooled experiments). Results are given as geometric mean fluorescence intensities (gMFI) with each dot representing the result from one mouse. The columns show the mean values +/- SD; unpaired student's t-test.
